# Evidence corroborates identity of isolated fossil feather as a wing covert of *Archaeopteryx*

**DOI:** 10.1038/s41598-020-65336-y

**Published:** 2020-09-30

**Authors:** Ryan M. Carney, Helmut Tischlinger, Matthew D. Shawkey

**Affiliations:** 1 Department of Integrative Biology, University of South Florida, 33620 Tampa, FL USA; 2 Tannenweg 16, 85134 Stammham, Germany; 3 Evolution and Optics of Nanostructures Group, Department of Biology, University of Ghent, 9000, Ghent, Belgium

**Keywords:** Palaeontology, Zoology

## Abstract

The historic fossil feather from the Jurassic Solnhofen has played a pivotal but controversial role in our evolutionary understanding of dinosaurs and birds. Recently, a study confirmed the diagnostic morphology of the feather’s original calamus, but nonetheless challenged the proposed identity as an *Archaeopteryx* covert. However, there are errors in the results and interpretations presented. Here we show that the feather is most likely an upper major primary covert, based on its long calamus (23.3% total length) and eight other anatomical attributes. Critically, this hypothesis is independently supported by evidence of similar primary coverts in multiple specimens of *Archaeopteryx*–including from the same fossil site and horizon as the isolated feather. We also provide additional insights, such as an updated colour reconstruction of the entire feather as matte black, with 90% probability. Given the isolated nature of the fossil feather, we can never know the anatomical and taxonomic provenance with 100% certainty. However, based on all available evidence, the most empirical and parsimonious conclusion is that this feather represents a primary covert from the ancient wing of *Archaeopteryx*.

## Introduction

**Figure 1:**
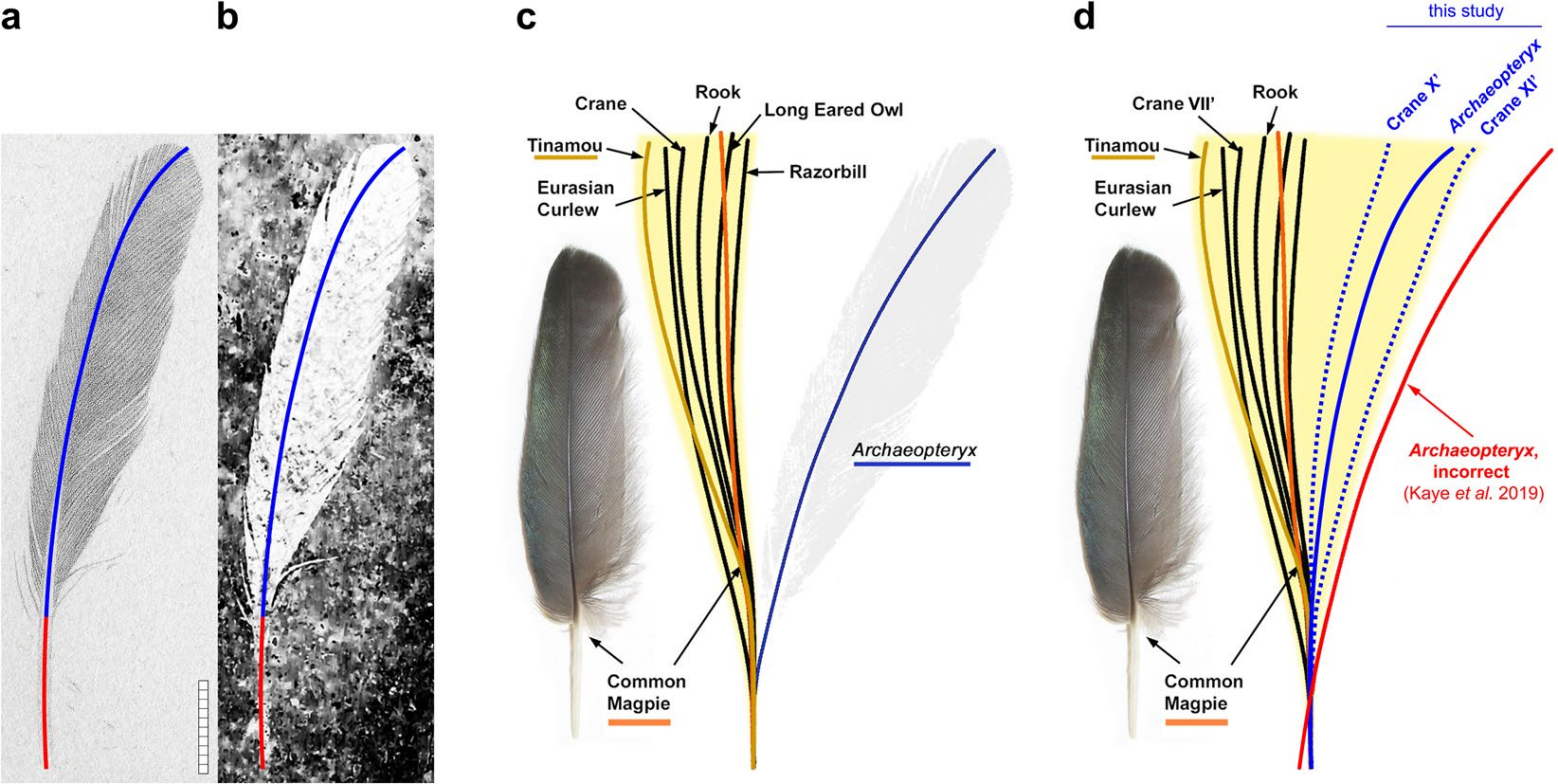
Centerlines of the isolated fossil feather and modern upper major primary coverts (UMPCs) from Kaye *et al*. 2019^8^. (**a**) Modified from von Meyer 1862: Plate VIII, Fig. 3^3^. (**b**) Laser-stimulated fluorescence image of MB.Av.100, modified from Kaye *et al*. 2019: Fig. 1^8^. In (**a**) and (**b**), the centerline comprises the calamus (red) and rachis (blue), and the feather is oriented so that calamus endpoints are vertically aligned. Images are reversed to match (**c**). Scalebar: 1 cm. (**c**) Reproduced from Kaye *et al*. 2019: Fig. 3^8^. The modern UMPCs presented here exhibit a strong anterior bend (leftward, towards the leading edge) near the calamus-rachis junction, creating an inflection point (S-curve) in the centerline. Note the yellow zone, representing the purported range of modern centerlines. (**d**) Modified figure from (**c**), including a more representative range of modern centerline morphologies (Common Crane X’ and XI’ from Supplementary Fig. S6, oriented so that calamus endpoints are vertically aligned). The correct *Archaeopteryx* centerline from (**a**,**b)** is overlaid as a solid blue line. This centerline was also overlaid onto the incorrect centerline to replicate the proximal end (red), revealing an alignment error. Note the substantial discrepancy between the correct and incorrect *Archaeopteryx* centerlines, the former of which now falls within the range of these modern primary coverts (yellow zone).

Renowned as the first fossil feather ever known^1–3^, the isolated feather specimen has played an important scientific role since its discovery in 1861^4,5^ (Fig. 1a, Supplementary Figs. S1, S2; MB.Av.100, BSP 1869 VIII 1). Seemingly misplaced in time–a modern-looking feather plucked from the Jurassic limestone–this prehistoric plume rewrote our understanding of the evolutionary history of birds and their dinosaur ancestors. This “Urfeder” (first feather) was the holotype of *Archaeopteryx lithographica*^2^, the archetypal Urvogel (first bird), until the feather was replaced by the more diagnostic London specimen^6^ (neotype, NHMUK PV OR 37001). Indeed, given its dissociation from any skeletal context, the isolated feather has always been in dispute^4,5^: where on the body did it come from, and is it from the same animal as the *Archaeopteryx* skeletons? Soon after the feather was unearthed, Owen^7^ cautioned against confusing anatomical variation with taxonomic affiliation, as this could lead to the erroneous conclusion that “the impression of a second feather differing greatly in its shape and proportions […] would represent a distinct species in Palaeontology”. In a recent study, Kaye *et al*.^8^ conflate such issues of anatomy and taxonomy, and challenge the proposed identity of the isolated feather as an upper major primary covert (UMPC) of *Archaeopteryx*^9^. They go even further in their press release, claiming with certainty that the “First discovered fossil feather did not belong to iconic bird *Archaeopteryx*”^10^. The three key arguments in Kaye *et al*. 2019^8^ are identified (boldface) and refuted as follows:

### “This ‘S-shaped’ centerline described here for the first time, appears to be a defining characteristic of primary coverts across a very broad range of modern species…”

The form and function of this S-shaped centerline had already been described elsewhere, however^11–14^. Specifically, a sigmoid curvature can cause the rachis of a primary covert to diverge anteriad and overlay at least one additional primary feather, for greater support during downstroke (Supplementary Figs. S3–S5). Critically, these previous studies also noted that the presence and degree of this S-curve is highly variable across species, and especially along the UMPC tract. The latter is not adequately considered in Kaye *et al*. 2019^8^, which only includes strongly S-shaped centerlines that are not representative of the overall morphological diversity, and thus do not provide for a valid comparison. For example, their “Crane” trace (Fig. 1c, below) appears to have been based on the most extreme S-curve (feather VII’) in the photograph of UMPCs from the Common Crane (*Grus grus*)^15^ (Supplementary Fig. S6). Conversely, the Common Crane feathers X’ and XI’ exhibit no S-curve at all, and instead curve posteriad towards the trailing edge (Fig. 1d: dotted blue centerlines). Plotting the full morphological range from the feather tract of this specimen alone more than doubles the area covered by modern feathers (Fig. 1d: yellow zone). Nearly half (46%, 11/24) of the species measured by Kaye *et al*.^8^ had one or more UMPCs with no S-shaped centerline^15,16^ (e.g., Supplementary Figs. S6–S8). Not only are not all UMPC feathers S-shaped, but not all S-shaped feathers are UMPCs: such centerlines can also be present in primaries, secondaries, upper major secondary coverts, under wing coverts, and alular feathers^15,16^.

### “…the isolated feathers [sic] centerline is a large departure from modern primary coverts”

However, their “*Archaeopteryx*” centerline is incorrect and cannot be reproduced from the paper’s results (Fig. 1c,d). In Fig. 1d, we overlay their original figure with a corrected centerline (solid blue) reconstructed from their laser-stimulated fluorescence image (LSF; Fig. 1b) and von Meyer’s 1862^3^ mirror trace (Fig. 1a; see also below). This new centerline is substantially different from that originally presented. Correcting for this error–and that of omission, above–completely eliminates the purported large departure of the fossil feather centerline, which now falls within the range of the selected modern species.

### Compared to secondary feathers in the Berlin specimen, “significant foreshortening of the isolated feather does not support its association with *Archaeopteryx*”

This argument is problematic for three reasons. First, the comparison is not appropriate given that secondaries had already been ruled out based on the aspect ratio of the isolated feather^9^. Second, this interpretation conflates the anatomical and taxonomic identities in an overly restrictive manner: just because the isolated feather is inconsistent with the secondaries of *Archaeopteryx* does not mean that the feather does not belong to *Archaeopteryx*–it could simply be a different type of feather. Indeed, while Kaye *et al*.^8^ state that “the isolated feather is not conformal to known *Archaeopteryx* specimens as a primary, secondary or tail feather”, they overlook impressions of other relevant feather tracts present in multiple specimens of *Archaeopteryx*. Third, the study’s alternative taxonomic hypothesis is a hypothetical undescribed dinosaur, a position that circumvents the burden of proof and cannot be falsified.

To best elucidate the nature of this feather, we must rely on an inferential “consilience of inductions”^17–19^–convergence among the independent classes of available evidence. Here we propose the following heuristic framework:

I. **What** ***anatomical identity*** **is most supported?**
  We evaluate the isolated feather across nine attributes, particularly the relative calamus length.
II. **What** ***taxonomic identity*** **is most supported?**
  We discuss the provenance, classification, and biological contexts relevant to the isolated feather.
III. **Are feathers of the** ***anatomical identity*** **preserved in fossils of the** ***taxonomic identity*?**
  We test these two hypotheses against all known skeletal specimens of *Archaeopteryx*.

## Results and Discussion

### Anatomical identity

#### Relative calamus length

Pennaceous feathers have a centerline (central shaft) that consists of a rachis distally and a calamus (quill) proximally (Fig. 1a). The rachis anchors the barbs that comprise the leading and trailing vanes, whereas the calamus is embedded within the skin. In both modern and Mesozoic birds^20^, the calamus inserts into a follicle and is attached to connective tissue and muscle that stabilize and control the feather^21,22^ (Supplementary Figs. S9, S10). Unlike the essentially solid and occasionally pigmented rachis, the calamus is a thin-walled and somewhat transparent hollow tube that is almost never pigmented^21^. These traits explain the relatively faint preservation of the fossil calamus as initially observed and illustrated by von Meyer^3^, and which has since become unobservable under visible and UV light^23^ (Supplementary Information: *11. Calamus preservation*).

Using LSF, Kaye *et al*.^8^ confirmed the morphology of the missing elongate calamus as matching that of von Meyer’s original description^3^ (Fig. 1a,b). This finding corroborates, rather than challenges, the proposed identity of the isolated feather–as an upper major primary covert (UMPC)^9^–which was based on that same original description. Von Meyer^3^ measured the straight-line lengths of the entire feather as 69 mm and the vaned portion as 54 mm, and the 15 mm difference was subsequently used for an estimate of relative calamus length (22%, 15/69 mm)^9^. Here, more accurate and precise measurements are obtained directly from a high-resolution digital scan of von Meyer’s 1862^3^ feather (Fig. 1a), originally traced at actual size using a drawing mirror^24^. Measuring from the lowermost barbs (which demarcate the calamus and rachis^21^), this yields a relative calamus length of 23.3% (16.4/70.3 mm). These measurements are congruent with those of the LSF image^8^ (Fig. 1b). See also Supplementary Information: *12. Calamus measurements* and Supplementary Fig. S11.

**Figure 2:**
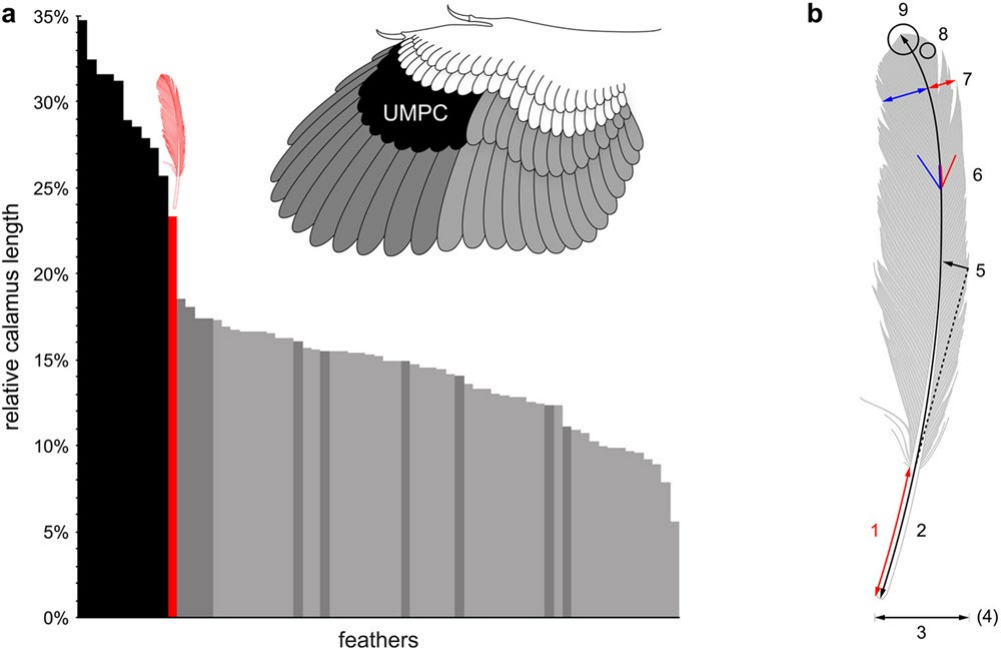
(**a**) Relative calamus lengths of modern feathers and the isolated fossil feather (red), in descending order. In both the plot (*Gallus*) and wing (*Archaeopteryx*), black represents UMPCs, dark grey represents primaries, and light grey represents secondaries and upper major secondary coverts (UMSCs), as well as rectrices (tail feathers) and alulars (not present in *Archaeopteryx*). UMPCs are statistically significantly longer than all other tracts. Inset: anatomical reference illustrating dorsal view of the left wing of *Archaeopteryx* (modified from Carney *et al*. 2012^9^; based on Wellnhofer 2009^4^, 2008^5^). Note that there are 12 primaries reconstructed in *Archaeopteryx* and 10 primaries in *Gallus*. See Supplementary Fig. S12 for anatomical reference of *Gallus* wing and the relative calamus lengths grouped by tract. (**b**) Schematic of the enumerated anatomical attributes.

When this relative calamus length is compared with those of modern feathers (*n* = 66; chicken, *Gallus gallus domesticus*)^21^, the closest matches are the UMPCs–particularly the distal members such as feather IX’ (25.7%, 18/70 mm; Fig. 2a, Supplementary Fig. S12). This similarity is reflected in Table 1, attribute #1. Among these modern feathers, the relative calamus lengths of the UMPC tract (*n* = 10) are found to be significantly longer than those of the five other tracts (*P*-values < 0.0001 for combined and all pairwise comparisons with the UMPC tract; Supplementary Fig. S12b). The extraordinarily long calami of UMPCs function to support the primaries during downstroke^13,25^.

**Table 1:** Comparison of anatomical attributes shared by the isolated fossil feather and modern feather tracts. Rows ranked by level of support. General consistency between the isolated feather and a given feather tract is designated “yes”, “no”, or “maybe” (occasionally) (see Supplementary Information: *15. Anatomical attributes*). Designations are primarily based on Lucas & Stettenheim 1972^21^ and phylogenetically broad surveys of online feather atlases^15,16^. Superscript denotes additional references for individual attributes (column headings), as well as previous hypotheses of anatomical identity (boldface row headings). Abbreviations: UMPC, upper (dorsal) major primary covert; uMPC, under (ventral) major primary covert; UMSC, upper major secondary covert.

feather tract	1. Relative calamus length	2. Length	3. Width	4. Aspect ratio	5. Lateral curvature^56,73^	6. Barb angle^74,75^	7. Vane asymmetry^56,57,73,76,77^	8. Vane closure	9. Angled distal tip^57^
**UMPC**^9^	**yes**	**yes**	**yes**	**yes**	**yes**	**yes**	**yes**	**yes**	**yes**
uMPC	no	**yes**	**yes**	**yes**	**yes**	**yes**	**yes**	**yes**	**yes**
**primary, distal**^57,78^	maybe	maybe	maybe	maybe	**yes**	**yes**	**yes**	**yes**	**yes**
primary, proximal	maybe	maybe	maybe	maybe	**yes**	**yes**	**yes**	**yes**	**yes**
**secondary**^4,5,56,57^	no	maybe	maybe	maybe	**yes**	**yes**	**yes**	**yes**	**yes**
UMSC	no	maybe	maybe	maybe	**yes**	**yes**	maybe	**yes**	maybe
rectrice, outer	no	maybe	maybe	maybe	maybe	**yes**	**yes**	**yes**	maybe
rectrice, inner	no	maybe	maybe	maybe	no	maybe	no	**yes**	maybe
**contour**^8^	no	no	**yes**	no	no	no	no	no	no

#### Anatomical attributes

Including the diagnostic relative calamus length, all nine anatomical attributes of the isolated feather are most consistent with an identity as a UMPC, to the exclusion of all other candidate feather tracts. These attributes include feather length, width, and aspect ratio, along with five attributes related to aerodynamics: lateral curvature, barb angle, vane asymmetry, vane closure, and angled distal tip (Fig. 2b). Table 1 summarizes general designations for each tract and attribute based on modern feathers^15,16,21^ (Supplementary Information: *15. Anatomical attributes*). Furthermore, attributes of the isolated feather that support it being a *distal* member of the UMPC tract include the relative calamus length being more similar (as mentioned above), vanes that are relatively asymmetric, and the angled distal tip; lack of an S-shaped centerline is also more consistent with distal UMPCs in some modern birds (e.g., compare these traits in distal vs. proximal members in Supplementary Fig. S6)^9,11,15,16,21^.

If we consider all anatomical attributes aside from the relative calamus length, the next most-supported identity of the isolated feather would still be a primary covert, albeit from a ventral tract–specifically, an under major primary covert (uMPC). The isolated feather would remain inconsistent with all other tracts. Kaye *et al*.^8^ hypothesized that the fossil could represent a contour feather, which is the predominant type of tract that covers the body. However, contours can be eliminated as a possibility given that almost every anatomical attribute is inconsistent with that of the isolated feather (Table 1). In particular, contours characteristically have a short calamus and length, as well as symmetric barb angles and vanes^21,26^.

### Taxonomic identity

Kaye *et al*.^8^ state of the feather, “The possibility remains that it stems from a different feathered dinosaur that lived in the Solnhofen Archipelago”. Philosophically, we agree–that possibility will always remain, as we can never identify the isolated feather with absolute, 100% certainty. However, the existence of two possibilities does not necessitate that they are equally probable. We must consider the full scope of available evidence, in order to provide the critical context with which to ground our assumptions. Ultimately, is it *more probable* that the isolated feather belongs to *Archaeopteryx*, or to a different taxon?

#### Provenance

**Figure 3:**
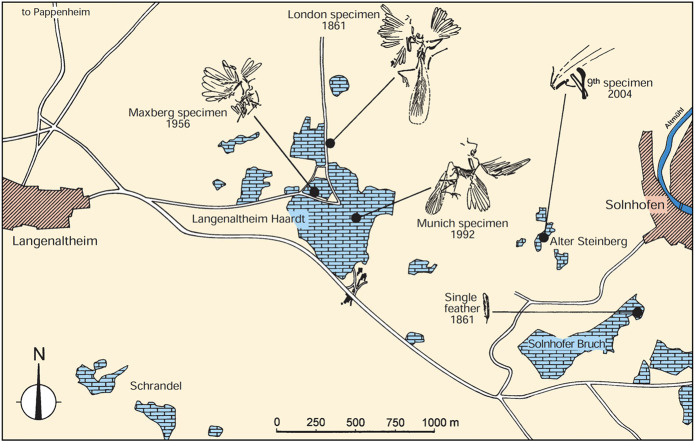
Map of the Solnhofen-Langenaltheim quarry district, illustrating locations of the isolated feather and the London (type), Maxberg, Munich, and Ottmann & Steil (9^th^) specimens of *Archaeopteryx*. Note that these five fossils are coincident in both space and time (see text). Reproduced by permission from Wellnhofer 2009^4^, 2008^5^: Fig. 5.98.

The most important evidence to consider is the overlooked fact that the isolated feather was actually found at the same fossil site as four specimens of *Archaeopteryx* (Fig. 3). These include the London (type), Maxberg, Munich, and Ottmann & Steil (9^th^) specimens^4,5^, all within ~750–2,200 meters of the isolated feather. These five fossils are also coeval, within the same *rueppellianus* horizon^27,28^ (Schweigert^29^ calculated 165 K years as the average duration for such Upper Jurassic ammonite horizons). Therefore, on the basis of this spatiotemporal proximity alone, the feather most likely originated from an *Archaeopteryx*.

#### Classification

The aforementioned four skeletons represent approximately one third of the 13 described *Archaeopteryx* specimens, and along with the Altmühl and Berlin specimens examined below, are all unequivocally classified as *Archaeopteryx*^4,5^. Overall, the taxonomy of the Urvogel specimens has been a complicated and contested topic–since their discovery, virtually every one of the skeletons has been referred to as a unique species and/or genus^4,5,30,31^. Recently, this includes controversial proposed assignments to a new species^32^, a new genus^33^, and even a different family^34^. It should be acknowledged, however, that these particular fossils represent three of the most fragmentary and poorly preserved specimens, and contain no clear feather impressions (Daiting, Mühlheim, and Haarlem specimens, respectively). Albeit sans the more recently described Mühlheim and Daiting (as well as Altmühl and Schamhaupten) specimens, all prior statistical analyses suggest that it is more parsimonious to interpret the skeletal differences as intraspecific variation, polymorphism, and/or ontogenetic scaling within *Archaeopteryx*^35–38^. Furthermore, all Urvogel fossils are clustered both geographically and temporally, within the Solnhofen Archipelago: only ~64 km apart^33^ and within maximum range estimates of 700 K to 1 M years^31,33^. For comparison, the temporal ranges of fossil avian species such as *Confuciusornis sanctus* and *Sapeornis chaoyangensis* are at minimum 5 M years each^39,40^.

#### Biological context

What about the alternative taxonomic hypothesis? It may be tempting and convenient to invoke some hypothetical undescribed dinosaur as the source of this isolated feather. However, it is important to keep in mind that the biodiversity within this small group of islands was finite, and represented but a brief snapshot in geologic time. While we are certainly not arguing against there being future discoveries, a great deal of this famous Lagerstätte’s biodiversity is already known–extraordinarily well-documented by ~600 species unearthed throughout hundreds of years of intense commercial quarrying and scientific efforts^4,5^. Among this voluminous and well-preserved fossil record, the other species of feathered dinosaurs–*Juravenator starki*^41^ and *Sciurumimus albersdoerferi*^42^–exhibit only the most ancestral class of feathers: unbranching monofilaments known as “protofeathers” (Stage I of Prum 1999^43^, Morphotype 1 and 2 of Xu *et al*. 2010^44^). Only *Archaeopteryx* specimens contain the most derived class of feathers to which the isolated feather belongs, with vanes that are pennaceous, closed, and asymmetric (Stage Va^43^, Morphotype 9^44^).

Even if such derived flight feathers were discovered in a new dinosaur species from these deposits–or if there were a permanent supraspecific reclassification of one of the 13 described Urvogels–the much higher abundance of *Archaeopteryx* specimens would still represent a much more likely affiliation for the isolated feather. And regardless, given that four of these unequivocal *Archaeopteryx* specimens were found at the same geologic time and place as the isolated feather, it is most probable that the isolated feather came from an *Archaeopteryx*.

### Fossil evidence

So far, we have demonstrated that the isolated feather is most likely a primary covert (anatomical identity), and most likely affiliated with *Archaeopteryx* (taxonomic identity). We now test these two hypotheses by examining all skeletal specimens of *Archaeopteryx* for evidence of primary coverts (consilience). See also Supplementary Information: *Fossil evidence*.

#### Altmühl specimen

**Figure 4:**
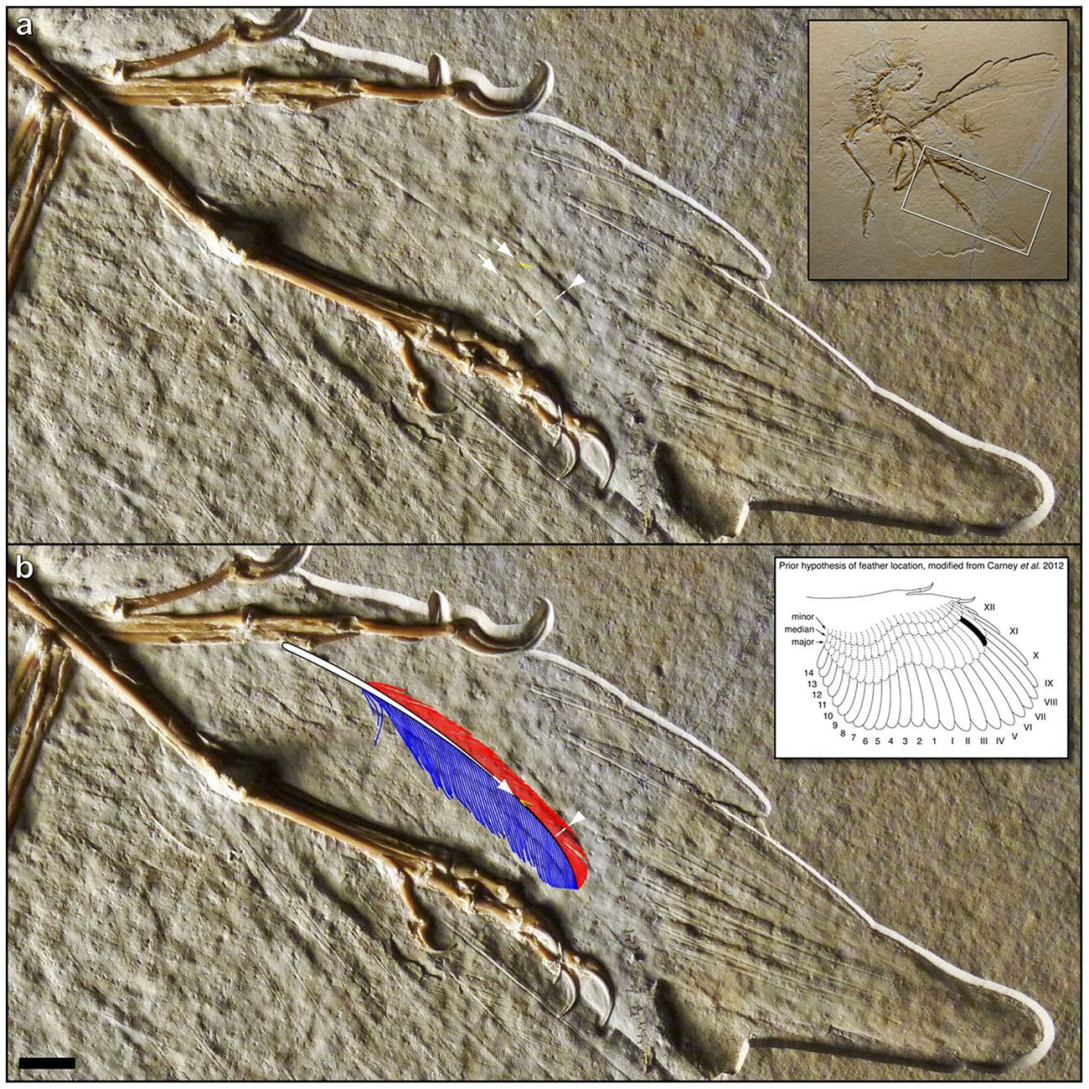
Altmühl specimen of *Archaeopteryx*, showing the dorsal surface of the right wing. (**a**) Key anatomical features denoted include two slightly curved rachis impressions (white arrows), two leading vane widths (small double arrows), the leading edge of the best-preserved UMPC (arrowhead), and a representative barb angle, which measures 25.1° (yellow lines; corresponding barb in isolated feather measures 25.2°; see Supplementary Fig. S15). Also note the posterior orientation of the UMPCs with respect to the manus and primaries, suggesting an absence of S-shaped centerlines. Inset: overview of specimen, denoting enlarged region. (**b**) Reconstruction of the isolated feather is overlaid at scale. Note the match in both size and shape to the underlying distal UMPC in the Altmühl specimen. Inset: black feather denotes prior hypothesis of the isolated feather’s approximate location, as a distal member of the UMPC tract^9^ (shown as a right wing to match that of the Altmühl specimen). Scale bar: 1 cm.

In 2014, Foth *et al.*^45^ described a new, 11^th^ specimen of *Archaeopteryx*, referred to as the “Altmühl specimen”^33^. Notably, this is the first and only known *Archaeopteryx* fossil to reveal a well-preserved *dorsal* surface of the wing–as evidenced by the overlapping pattern of primaries^45^, convex vanes with positive casts of barbs, absence of ventral furrows on rachises, and absence of elongate under covert barbs overlying the secondaries (cf. Berlin specimen, below). This right wing exhibits impressions of at least four UMPCs, approximately half the length of their respective primaries (Fig. 4; Foth *et al*. 2014: Extended Data Fig. 5b^45^).

Strikingly, the two best-preserved UMPCs are identical to the isolated feather in every observable attribute of size and shape, including barb angle (Fig. 4; Table 2). Barb angles on the distal half of one leading vane measure a mean of 25.2° (*n* = 5); the corresponding barb angles on the isolated feather measure a mean of 24.4° (*n* = 5) (Supplementary Fig. S15). The vanes are closed (#8) and asymmetric (#7), albeit each trailing edge is overlapped by the adjacent UMPC, inhibiting exact measurements of feather width. However, inferring from the leading vane widths and spacing of the UMPCs, as well as the widths and spacing of the primaries, the UMPC width (#3) and consequent aspect ratio (#4) are considered consistent with those of the isolated feather. The presence of an angled distal tip cannot be ascertained in the UMPCs (#9), nor can relative calamus length (#1) due to overlying median and minor covert tracts.

**Table 2:** Comparison of anatomical attributes shared by the isolated fossil feather and major primary coverts of *Archaeopteryx* specimens. Rows ranked by level of support. Consistency between the isolated feather and a given feather tract is designated “yes”, “no”, or “?” (not observable) (see Supplementary Information: *15. Anatomical attributes*). See Supplementary Table S1 for other tracts. Abbreviations: UMPC, upper (dorsal) major primary covert; uMPC, under (ventral) major primary covert.

feather tract (specimen)	1. Relative calamus length	2. Length	3. Width	4. Aspect ratio	5. Lateral curvature	6. Barb angle	7. Vane asymmetry	8. Vane closure	9. Angled distal tip
UMPC (Altmühl)	?	**yes**	**yes**	**yes**	**yes**	**yes**	**yes**	**yes**	?
uMPC (Berlin)	?	**yes**	**yes**	**yes**	**yes**	**yes**	**yes**	no	no
uMPC (London)	?	**yes**	?	?	**yes**	**yes**	?	?	?

This congruence corroborates the isolated feather’s approximate location as a distal member of the UMPC tract, as hypothesized by Carney *et al*. 2012^9^ (Fig. 4b: inset). The fact that the feathers are equivalent in size also suggests that the isolated feather may have originated from an individual approximately the same size as the Altmühl specimen, depending on the exact feather number. (Relative specimen sizes^4,5,45^: Berlin < Altmühl < London).

**Figure 5:**
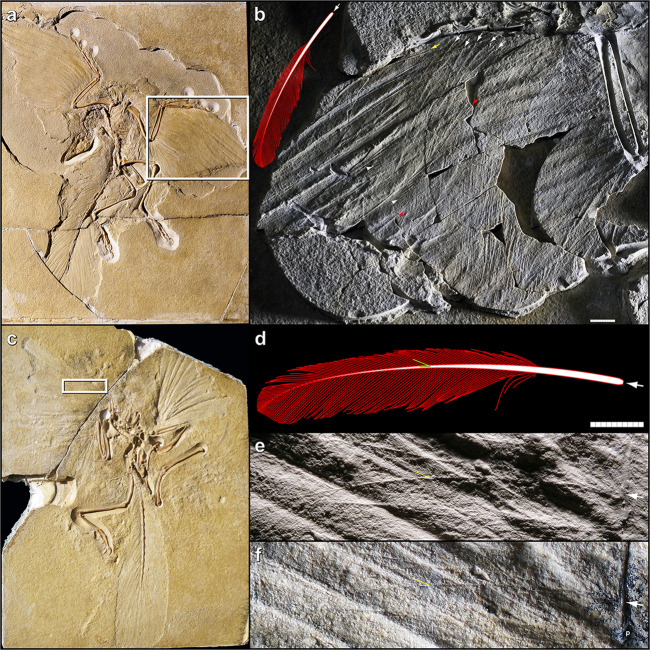
Under primary coverts in the Berlin and London specimens of *Archaeopteryx*. White arrows indicate proximal orientation of centerlines. (**a**) Berlin specimen (MB.Av.101), main slab. (**b**) Right wing region from white box in (**a**), from the counterslab under oblique lighting. Reconstruction of the isolated feather is overlaid at scale. White arrowheads denote the non-interlocking “split” barbs of open pennaceous vanes. Red arrow and arrowhead denote centerline of a uMPC, which exhibits a ventral furrow on the proximal end. Yellow arrow denotes S-shaped centerline in the penultimate primary. Note the orientation of the coverts, and their posterior divergence from the primaries. (**c**) London specimen, main slab (NHMUK PV OR 37001); reproduced by permission from Wellnhofer 2009^4^, 2008^5^. (**d**) Reconstruction of the isolated feather. Yellow lines indicate barb angle measured in the London specimen in (**e**); angle of the corresponding barb in the isolated feather measures 25.8°. (**e**) Left wing region from white box in (**c**), from a cast of the counterslab (image is reversed). Centerline is preserved as a positive cast. Yellow lines indicate a representative barb angle, which measures 24.6°. Shown at same scale as (**d)**. (**f**) Left wing region from white box in (**c**), from the original main slab under oblique lighting. Centerline is preserved as a negative impression. Negative impression of digit II phalanx 2 is denoted by “p”. Note the very close match in size, curvature, and barb angles between the isolated feather and primary coverts from both specimens. Scale bars: 1 cm.

While the entire UMPC centerlines are not directly observable, we can infer that they were not S-shaped, based on three observations. First, the UMPCs are angled too posteriorly (diagonally) from the manus. Such oblique orientation also causes the UMPCs to diverge from the primaries–and especially the penultimate primary, which is S-shaped (Fig. 4; see also^45,46^). Second, this posterior divergence is dissimilar to the anterior divergence of modern UMPCs from the primaries^47^ (e.g., Supplementary Fig. S3). Therefore, any dissimilarity with respect to modern S-shaped centerlines would be unsurprising. Third, this posterior divergence is similar to that of under primary coverts in both wings of the Berlin specimen, and those feathers clearly lack S-shaped centerlines (Fig. 5b). Elzanowski^48^ noted that those coverts’ “preserved, strongly diagonal position with respect to the primaries has yet to be explained”. Here we offer the explanation that the posterior divergence in both specimens may have strengthened the wing for flapping flight. Namely, such primary coverts–angled via calamus attachment and/or rachis curvature–would have supported the primaries during downstroke by crossing over them posteriad, instead of anteriad as in the case of S-shaped centerlines discussed above.

#### Berlin specimen

As mentioned above, the second most-supported anatomical identity of the isolated feather is a uMPC. Such uMPC centerlines in the Berlin specimen (MB.Av.101) were first illustrated in Dames 1884^49^ and Steiner 1918^50^, and subsequently illustrated, photographed, and described in Heinroth 1923^51^, Heilmann 1926^52^, de Beer 1954^53^, Helms 1982^54^, and Rietschel 1985^55^. Multiple tracts of primary coverts are visible on the main slab, and especially the counterslab, where they are less worn^4,5^ and preserved as positive casts (Fig. 5b). It is difficult to attribute some centerlines to distinct coverts^55^. Both slabs represent the ventral surface, as evidenced by the ventral furrows of rachises, pattern of feather overlap, and corresponding positive and negative casts of the barbs^4,5,48,54,55^. As noted by previous authors^49,54,55^, elongate “floating” barbs are apparent in coverts of the secondary and proximal primary feathers.

In the uMPCs, relative calamus lengths are not observable (#1), but attributes #2–7 are consistent with those of the isolated feather (Table 2). A total of at least seven uMPCs from both wings exhibit a rounded distal tip (#9) and/or open pennaceous vanes^55^ (#8; Fig. 5b: white arrowheads). Both an angled distal tip and vane closure are aerodynamic features that may not be present in modern uMPCs^15,16^, or that may appear in wing coverts later in ontogeny^21^.

Both wings have a well-preserved uMPC^52,55^ originating from the same approximate midpoint of the manus, with a ventral furrow in the rachis similar to that of the primaries (Fig. 5b: red arrow). Such furrows increase dorsoventral stiffness^56^, indicating a supportive function. As discussed above, the primary coverts from multiple tracts have a strongly posterior (diagonal) orientation with respect to the manus and primaries^48,54^ (Fig. 5b: white and red arrows), similar to the UMPCs in the Altmühl specimen, and which likely strengthened the wing. This posterior divergence is due to the centerlines’ angled attachments, as well as their curvature, which is consistent with that of the isolated feather but not with an S-curve. Conversely, a distinct S-shaped centerline is evident in the penultimate primary of both wings (Fig. 5b: yellow arrow), similar to the penultimate primary of the Altmühl specimen (above), and the distalmost, diminutive primary of some modern birds as well^15,16^.

#### London specimen

The London specimen of *Archaeopteryx* also preserves fine details of the ventral wing surfaces^4,5,7,50,53^ (Supplementary Fig. S14). In his classic monograph, de Beer^53^ remarked that the isolated feather’s “similarity to one of the shorter remiges is very great… the impressions in the British Museum specimen might well have been made by feathers identical with von Meyer’s feather”. On the left wing, he counted impressions of seven uMPCs, approximately half the length of their respective primaries. On the right wing, Steiner^50^ measured the lengths of two proximal uMPCs as 55 and 60 mm (identified as upper I’ and II’, albeit as under II’ and III’ per de Beer^53^; Supplementary Fig. S14c). All visible primary coverts exhibit a laterally curved or straight centerline, with no evidence of an S-curve. Notably, the most conspicuous centerline is indistinguishable from that of the isolated feather with respect to length (#2) and lateral curvature (#5) (Fig. 5d–f; Table 2). Tentatively, this centerline may represent the distal uMPC X’, based on a presumed attachment at the proximal half of digit II phalanx 2 (Fig. 5f: p; see also Supplementary Fig. S14b; Fig. 6.17a in Wellnhofer 2009^4^, 2008^5^). Barbs are visible on the leading vane of this feather in the counterslab, and to a lesser extent in the main slab (Fig. 5e,f). These barb angles are consistent with those of the isolated feather (#6; Fig. 5d) and primary coverts in the Altmühl and Berlin specimens. Presence of the remaining attributes cannot be determined in the uMPCs.

#### Anatomical attributes

Ultimately, we accept the dual hypotheses that the isolated feather is a UMPC of *Archaeopteryx*, given that the isolated feather is consistent with UMPCs of the Altmühl specimen in every observable anatomical attribute (Table 2). Most of the isolated feather’s attributes are also consistent with those of the Berlin and London specimens’ uMPCs–which serve as proxies given that uMPCs generally resemble their UMPC counterparts^21,47^. However, there is less overall support for the isolated feather being a uMPC, given that such feathers visible in the Berlin specimen exhibit open vanes and rounded distal tips. The centerline of the isolated feather is also thicker^3,8^ than those of the uMPCs observable in the Berlin and London specimens (Fig. 5), but is consistent with the robust centerlines of modern UMPCs^21,22^. The isolated feather is inconsistent with all of the other tracts in *Archaeopteryx* (Supplementary Table S1).

### Additional insights

#### Designation

There has been perpetual disagreement in the literature regarding which half is designated as the main slab (versus the counterslab): the Munich slab^4,5,8,9,53^ (BSP 1869 VIII 1) or the Berlin slab^23,57,58^ (MB.Av.100). Here, we propose that the Berlin slab be formally designated as the main slab, given 1). von Meyer’s^3^ original terminology (Supplementary Information: *21. Designation*), as well as the fact that the Berlin slab 2). has always contained much better-preserved traces of the feather^3,8,9,23^, 3). is thicker and 2.6X larger by area^3,24^, and 4). contains more positive remains of fossil material (i.e., *Saccocoma tenella* crinoids)^23^, which typically distinguish main slabs in Solnhofen fossils^30^ (Supplementary Figs. S1, S2). The darker trace on the Berlin slab, coupled with microstructural evidence, also indicates that the feather originated from the left wing of the animal (Supplementary Information: *22. Feather chirality*, Supplementary Fig. S16).

#### Preservation

The feather was most likely shed during moult, given the otherwise firm attachment of wing feathers, even post-mortem^4,5,57,59^. This same dissociation that obfuscates the feather’s identity is likely also responsible for the dark preservation, compared with that of the plumage in the skeletal specimens (taphonomy reviewed in Wellnhofer 2009^4^, 2008^5^). While Kaye *et al*.^8^ state that the isolated feather may be preserved as a film of manganese dioxide, Carney *et al*.^9^ had previously detected no such manganese, and instead interpreted this dark film to be a melanic organosulphur residue. This interpretation was subsequently supported by molecular evidence of melanin associated with such residues in other fossil and extant feathers, even when the melanosome structures themselves have completely degraded^60,61^. Thousands of melanosomes (not microbes, contra Moyer *et al*. 2014^62^–see Supplementary Information: *24. Melanosomes*) are observable in varying states of preservation within the isolated feather, and the melanin residue is ubiquitous throughout both vanes^9^. This pervasive residue darkens towards the distal tip on both slabs^9^ (Supplementary Figs. S1, S2), representing a subtle melanin concentration gradient^63^. Together, these modern results confirm von Meyer’s original 1862 hypothesis, that the darker tip was “caused by the original colouration”^3^.

#### Colouration

**Figure 6:**
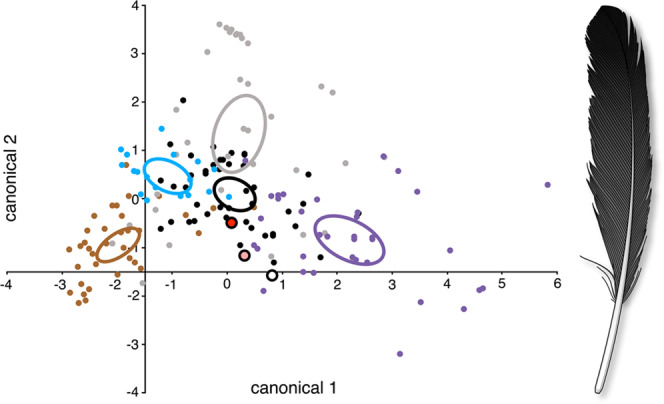
Feather colour reconstruction, based on quadratic discriminant analysis of melanosome morphologies from modern feathers representing various classes: brown (brown), grey (grey), iridescent (purple), matte black (black), and penguin type (blue). Ellipses represent 95% confidence bounds. Results from the isolated feather (MB.Av.100) represent measurements^9^ of melanosome imprints only (red, most reliable), imprints and 3D preservation combined (pink), and 3D preservation only (white). Canonical axes 1 and 2 are most strongly associated with aspect ratio and length, respectively^66^.

Melanosome morphology previously predicted the original colour of the isolated feather to be black^9^, based on statistical comparison with a dataset of melanosomes from extant birds^64,65^. Using a subsequently expanded dataset that includes iridescent melanosome morphologies^66^, our current reanalysis of the fossil feather’s melanosome imprints (*n* = 86) predicts that the black colouration was matte, with 90% probability (0% probability of iridescent; Fig. 6). Adding measurements from the three-dimensionally (3D) preserved melanosomes to the analysis yields a prediction of matte black with 85% probability (*n* = 108, 15% probability of iridescent), whereas analysis of the 3D melanosomes alone predicts iridescent with 79% probability (*n* = 22, 21% probability of matte black). This difference in results is due to diagenetic contraction of the 3D melanosomes^61,67^, a phenomenon which should have negligible effect on the melanosome imprints in the limestone matrix^61,68^. We therefore consider the imprint-only results to be the most reliable. Such matte black colouration is associated with the lower mean aspect ratio of these eumelanosomes (*n* = 86, 3.8 ± 0.1 SE), compared with the more elongate iridescent morphology^66^.

Thus, we clarify and refine our previous findings, by reconstructing the *entire* feather as *matte* black with a darker distal tip (contra the incorrect black and white reconstruction of Manning *et al*. 2013^69^–see Supplementary Information: *25. Colouration* and Supplementary Fig. S17). Such black colouration throughout the entire feather–including the non-visible (obscured) proximal region–is consistent with the observation that modern UMPCs “are most frequently whole-coloured and dark”^70^. As in modern feathers, this black pigmentation (especially at the distal tip) would have provided various structural^9^ and aerodynamic^71^ advantages to the wings of *Archaeopteryx*. The similar presence of dark remiges across Paraves (e.g., *Microraptor*^66^, *Caihong*^72^) suggests an important functional role for melanization in the evolution of dinosaur flight.

## Summary

The fossil record serves as life’s time capsule, albeit a vastly imperfect one. By virtue of the fossil feather’s isolated nature, we can never know the exact follicle or species from which the Urfeder originated with complete certainty. Rather, we must rely on a framework of consilience: the convergence among the independent classes of available evidence. What are the most likely anatomical and taxonomic identities, and are both of these hypotheses supported by the fossil data?

Anatomically, lack of an S-shaped centerline does not preclude the isolated feather from being a UMPC. Comparing the corrected fossil feather centerline with a more representative range of extant morphological diversity eliminates all purported disparity. The isolated feather had a proportionately long calamus–an attribute diagnostic of UMPCs, which have the greatest relative calamus length of any feather tract. All eight of the other anatomical attributes corroborate the hypothesis that the isolated feather is a UMPC, to the exclusion of all other feather tracts.

With respect to taxonomic identity, the most critical piece of evidence is that the feather specimen came from the same fossil site and horizon as four *Archaeopteryx* skeletons, including the type specimen. Furthermore, within the extraordinarily well-documented and spatiotemporally limited Solnhofen Archipelago, only *Archaeopteryx* specimens exhibit such a highly derived feather morphotype. In the future, even if a new feathered dinosaur species were revealed (or reclassified) from these deposits, the present fossil would still most likely represent a feather from the much more coincident and abundant *Archaeopteryx*.

Testing these anatomical and taxonomic hypotheses against the fossil data, the isolated feather is conformal to the primary coverts of *Archaeopteryx*. Specifically, the isolated feather is identical in size, shape, and barb angles to UMPCs in the Altmühl specimen, and to a lesser extent, to uMPCs in the Berlin and London specimens. None of these feathers exhibit any indication of an S-shaped centerline. All other tracts in *Archaeopteryx* are inconsistent with the isolated feather.

Ultimately, supported by all of the anatomical and taxonomic evidence, independently confirmed by close morphological connections to multiple skeletal specimens, the most empirical and parsimonious conclusion is that the isolated feather represents a primary covert of *Archaeopteryx*. Additionally, we recommend that the Berlin slab be designated as the main slab, reveal that the feather originated from the left wing, and reconstruct the original feather colour as entirely matte black.

## Methods

The von Meyer 1862: Plate VIII, Fig. 3^3^ was digitally scanned along with a mm ruler for validation at 1,200 dpi using an HP OfficeJet 5255 flatbed scanner. Linear, curvilinear, and area measurements of the isolated fossil feather and slabs^3,24^ were taken in Adobe Illustrator CS6 using the Telegraphics Patharea filter version 1.2b3 (http://telegraphics.com.au/sw/product/patharea). The isolated feather reconstruction was based on Fig. 4 from Carney *et al*. 2012^9^–originally recreated in Adobe Illustrator CS4 based on photographs of the von Meyer reconstruction^3^–with modifications made in Adobe Illustrator CS6 based on the high-resolution scan of the von Meyer reconstruction^3^, photographs^9,57^ and the LSF image^8^ of the Berlin slab (MB.Av.100), and ultraviolet light photographs of the Munich slab (BSP 1869 VIII 1; e.g., Supplementary Fig. S11). Barb angles for the skeletal specimens were defined using the Line Segment Tool in Adobe Illustrator CS6 (blindly, without the isolated feather overlay). In the Altmühl specimen, barbs are preserved as positive (convex) casts; for better precision the barb angles were defined using the narrower negative impression of the space between adjacent barbs. All barb angles were subsequently measured using ImageJ version 1.52k (https://imagej.nih.gov/ij/). Relative calamus lengths of modern feathers (*n* = 66) were calculated using published measurements^21^ from a male Single Comb White Leghorn Chicken (*Gallus gallus domesticus*) at least one year old, and analyzed using JMP Pro version 14.0 (SAS Institute Inc.). Pairwise comparisons among the six feather tracts were made using one-way ANOVA with Tukey-Kramer HSD. Each tract was found to be normally distributed using the Shapiro-Wilk test, with the exception of the rectrices (*n* = 8, *P*-value = 0.0017), and when the five tracts of non-UMPC feathers were combined (*n* = 56, *P*-value = 0.0208). Therefore, values from these five tracts combined were compared with those of the UMPC tract (*n* = 10) using the one-tailed Wilcoxon exact test (sum of rank scores = 615). All variances were found to be equal. Melanosome measurements from the isolated feather were taken from Carney *et al*. 2012: Supplementary Table S1^9^. For details on the quadratic discriminant analysis, see Li *et al*. 2012^66^.

## Supplementary information

Supplementary Information.
