# Supplementary material for: Evidence corroborates identity of isolated fossil feather as a wing covert of Archaeopteryx
Source: Sci Rep. 2020 Sep 30;10:15593. doi: 10.1038/s41598-020-65336-y (PMC7528088; doi:10.1038/s41598-020-65336-y)
Supplement: Supplementary file 1 — Supplementary Information. [file 41598_2020_65336_MOESM1_ESM.pdf]

Supplementary Information

for

Evidence corroborates identity of isolated fossil feather as a wing covert of *Archaeopteryx*

Ryan M. Carney*, Helmut Tischlinger, Matthew D. Shawkey
*ryancarney@usf.edu

Introduction

1. Figure S1. Isolated fossil feather: Berlin slab 2
2. Figure S2. Isolated fossil feather: Munich slab 3
3. Figure S3. Centerlines: Pycraft 1910 4
4. Figure S4. Centerlines: Jeikowski 1971 4
5. Figure S5. Centerlines: Stegmann 1962, Wray 1887 5
6. Figure S6. UMPCs: crane 6
7. Figure S7. UMPCs: quail 7
8. Figure S8. UMPCs: various 7

Anatomical identity

9. Figure S9. Calami: chicken 8
10. Figure S10. Calami: schematic 8
11. Calamus preservation 9
12. Calamus measurements 9
13. Figure S11. Comparative images of the isolated fossil feather 10
14. Figure S12. Relative calamus lengths by tract 11
15. Anatomical attributes 12

Fossil evidence

16. Supplementary Table S1 13
17. Other *Archaeopteryx* specimens 13
18. Figure S13. Maxberg specimen 14
19. Figure S14. London specimen: Owen 1863 15
20. Figure S15. Barb angle measurements 16

Additional insights

21. Designation 17
22. Feather chirality 17
23. Figure S16. Barbule microstructure 18
24. Melanosomes 19
25. Colouration 19
26. Figure S17. Reconstructions of the isolated fossil feather 19

References

References 20

Introduction

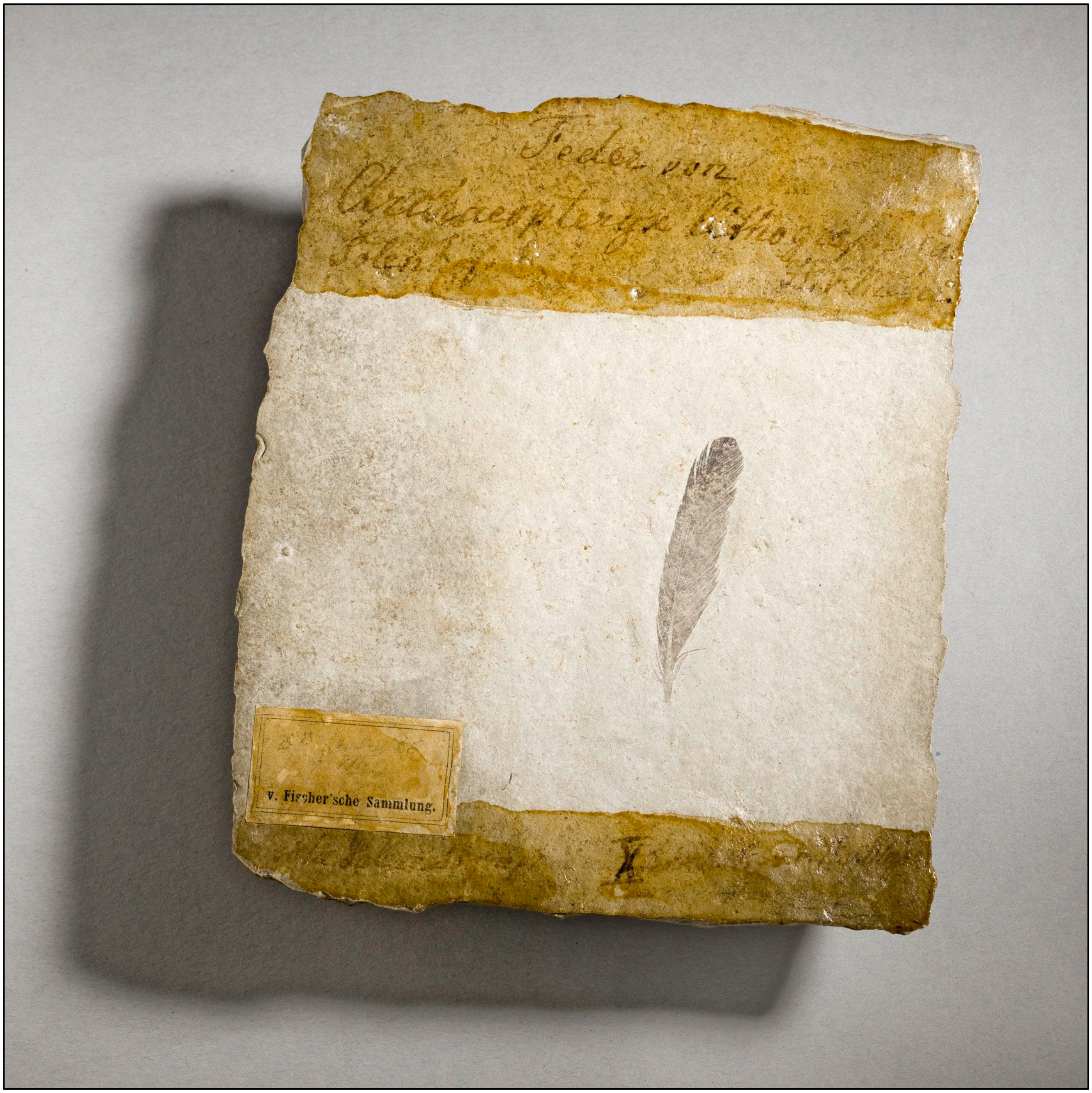

1. Supplementary Figure S1. Berlin slab of the isolated fossil feather (MB.Av.100). Proposed herein to be designated as the main slab. Housed in the Museum für Naturkunde in Berlin, Germany. Image courtesy of the Museum für Naturkunde.

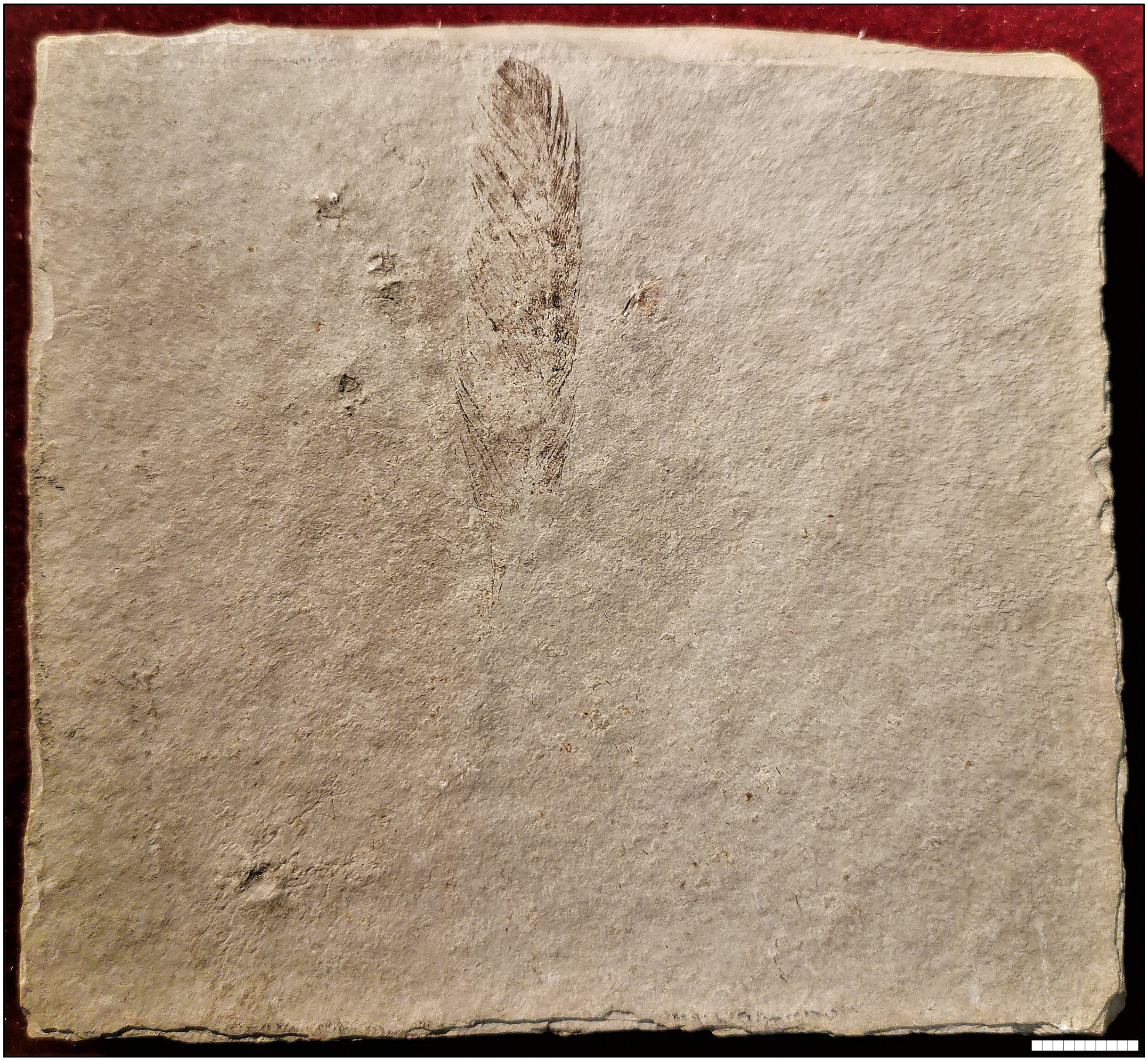

2. Supplementary Figure S2. Munich slab of the isolated fossil feather (BSP 1869 VIII 1). Proposed herein to be designated as the counterslab. At the bottom left corner and to the left of the feather are negative impressions of crinoid, *Saccocoma tenella*. Such negative impressions are typical of counterslabs of Solnhofen fossils. Housed in the Bavarian State Collection for Palaeontology and Geology in Munich, Germany. Scalebar: 1 cm.

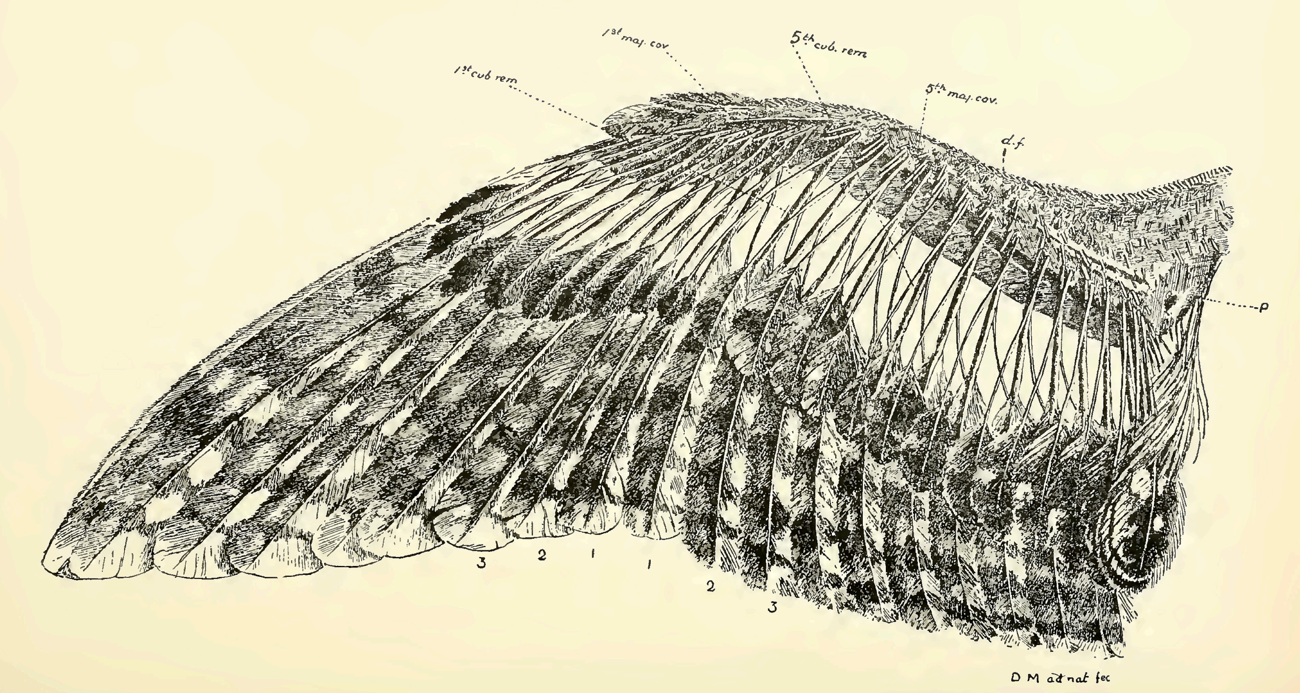

Ill. 37.—Wing of a Long-eared Owl, Showing the Pointed Type of Wing Associated with Strong Powers of Flight

3. Supplementary Figure S3. Reproduced from Pycraft 1910[1]. Dorsal surface of the left wing with much of the vanes removed, illustrating the S-shaped centerlines of the upper major primary coverts (UMPCs), and to a lesser extent, of the upper major secondary coverts (UMSC). Note the resulting anterior divergence of the UMPCs (i.e., distally, towards their leading edge), which cross multiple primaries. This is distinctly different from the posterior divergence of the UMPCs in the Altmühl specimen of *Archaeopteryx* (Fig. 4).

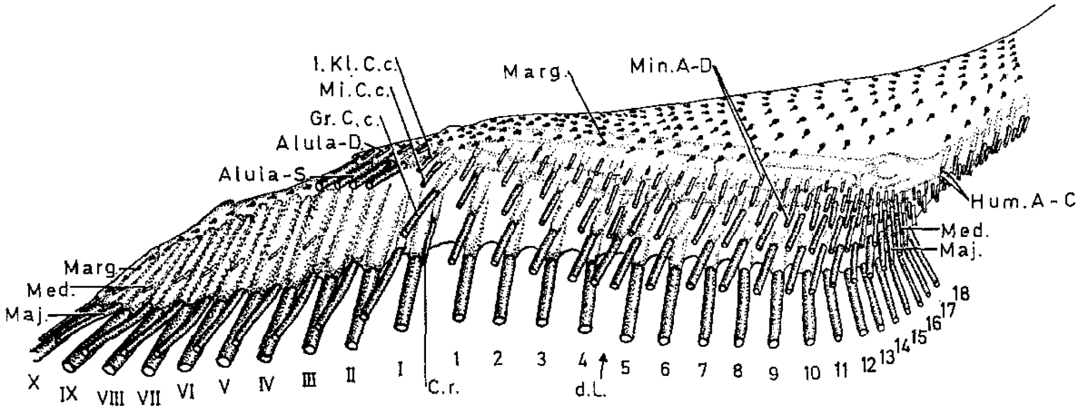

Abb. 5. Insertion der Federn der Flügeloberseite (linker Flügel).
Federn kurz abgeschnitten; unter der Haut verborgene Spulen sowie Skelett durch feine Punktierung angedeutet. Randfedern durch Punkte symbolisiert, an denen ein Strich die Richtung des Schafts anzeigt. I—X = Handschwingen; 1—18 = Armschwingen; d. L. = diastataxische Lücke; Alula-S. = Alulaschwungfedern; Alula-D. = Obere Aluladeckfedern; Gr., Mi., 1. Kl. C. c. = Großes, Mittleres und 1. Kleines Carpal covert; C. r. = Carpal remex; Hum. A—C = Obere Humeralfedern der Reihen A bis C; Maj. = Große Obere Deckfedern; Marg. = Obere Randfedern; Med. = Mittlere Obere Deckfedern; Min. A—D = Kleine Obere Deckfedern der Reihen A bis D.

4. Supplementary Figure S4. Reproduced from Jeikowski 1971[2]. Dorsal surface of the left wing with the vanes and most of the rachises removed. Note the close and parallel attachment of the UMPC calami with the respective primary calami, and the S-curve which causes the UMPC rachises to diverge anteriad and cross over the respective primary rachises.

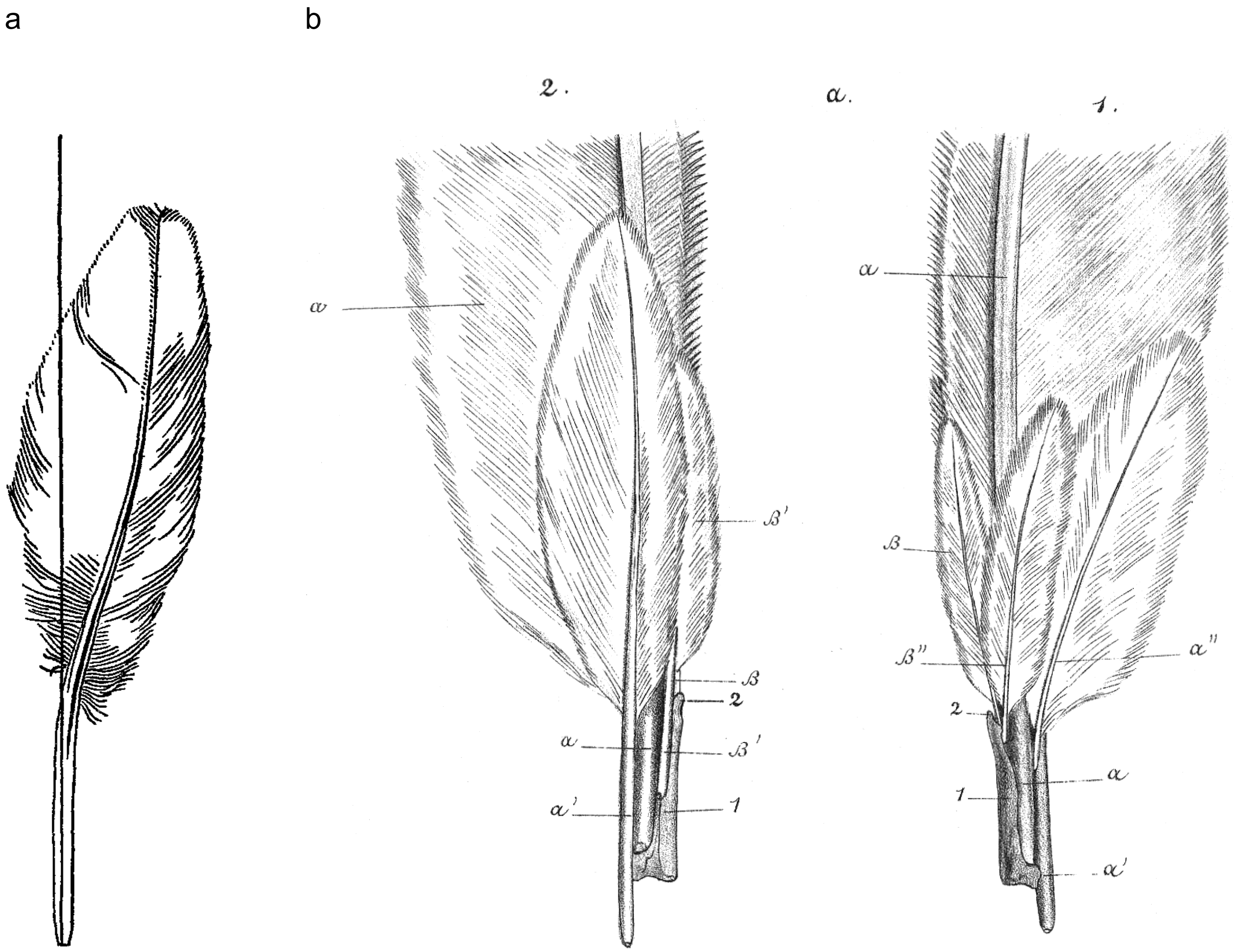

Plate XXXI.

a. The distal phalanx of digit II. of the wing of the Barn-Owl, with the attached predigitals and their coverts, showing the remicle and its relations.

1. Ventral view. 2. Dorsal view.

α. Predigital 1. *β*. Predigital 2 (remicle).
α'. Dorsal tectrix major to *α*.
α''. Ventral tectrix major to *α*.
β'. Dorsal tectrix major to remicle.
β''. Ventral tectrix major to remicle.
1. Phalanx 2 of digit II.
2. Fused phalanx 3 of digit II.

5. Supplementary Figure S5. Centerlines. (**a**) Reproduced from Stegmann 1962[3], illustrating a UMPC with a strong S-shaped centerline. (**b**) Reproduced from Wray 1887[4], illustrating the dorsal view of two UMPCs (*a'*, *β'*) from the Barn Owl (*Tyto alba*). Note that not all UMPC centerlines are S-shaped. Also note the relatively long calami of the UMPCs compared with those of the under major primary coverts (uMPCs; *a''*, *β''*), primary (*a*), and remicle (*β*).

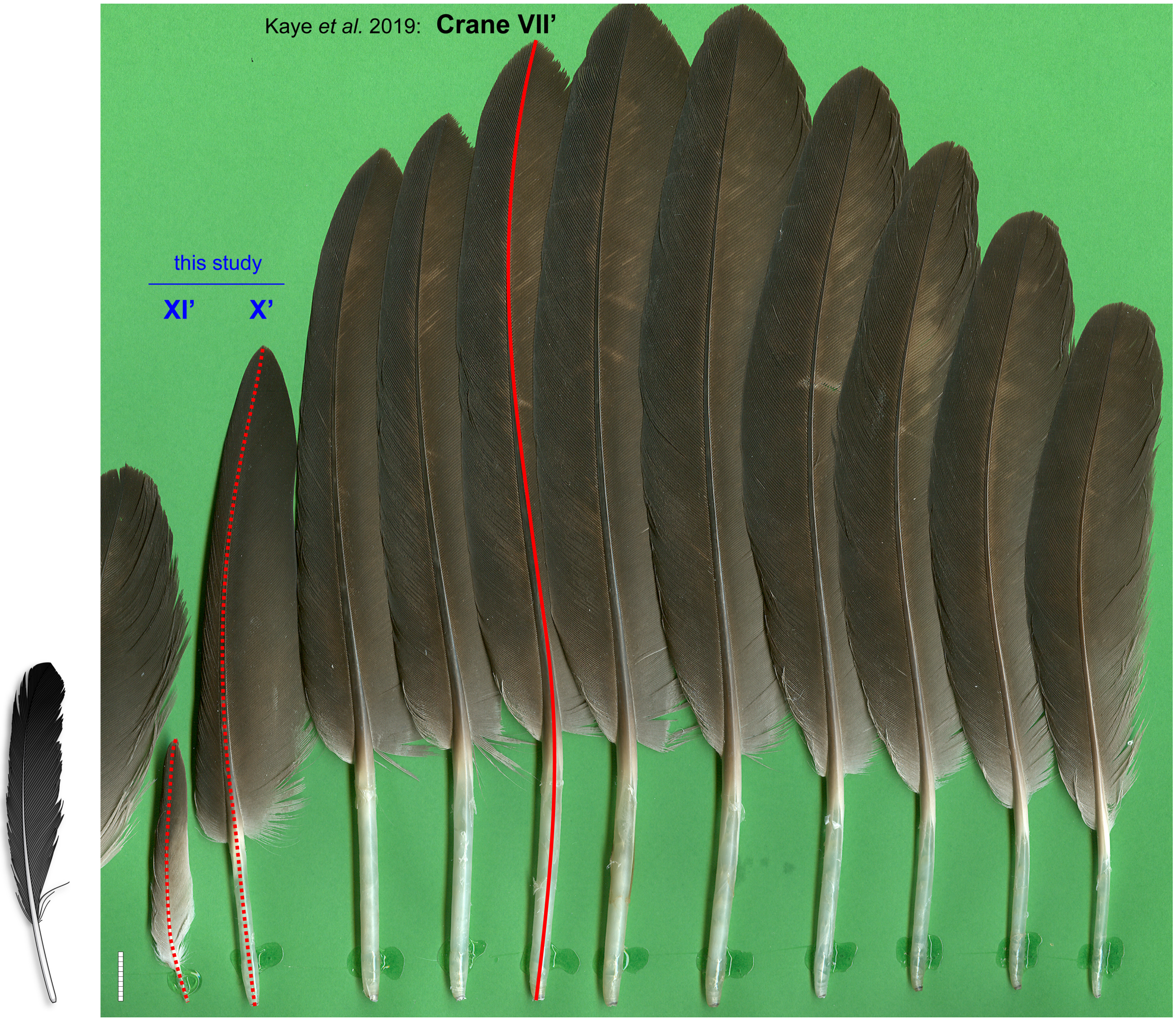

6. Supplementary Figure S6. UMPC tract from the immature Common Crane (*Grus grus*) specimen studied in Kaye *et al.* 2019[6]. Centerlines for feathers XI' and X' are reconstructed here as dashed red lines, and used for Fig. 1d. The solid red centerline is reconstructed from Kaye *et al.* 2019: Fig. 3[6] (reproduced in Fig. 1c herein), and overlaid onto these feathers to determine its identity (VII'). The isolated fossil feather at far left is shown to scale. Note that the curvature (and size) of the isolated fossil feather falls between those of feathers XI' and X'. Conversely, all three feathers are distinctly different from the superlative S-curve of feather VII' presented by Kaye *et al.* 2019[6]. Also note the angled distal tips, especially in the more distal members of the tract (towards the left). Original image used with the permission of Stephan Schubert; lower-resolution version hosted at http://www.vogelfedern.de/kr_01g.htm[5]. Image is reversed to match the aforementioned figures. Scale bar: 1 cm.

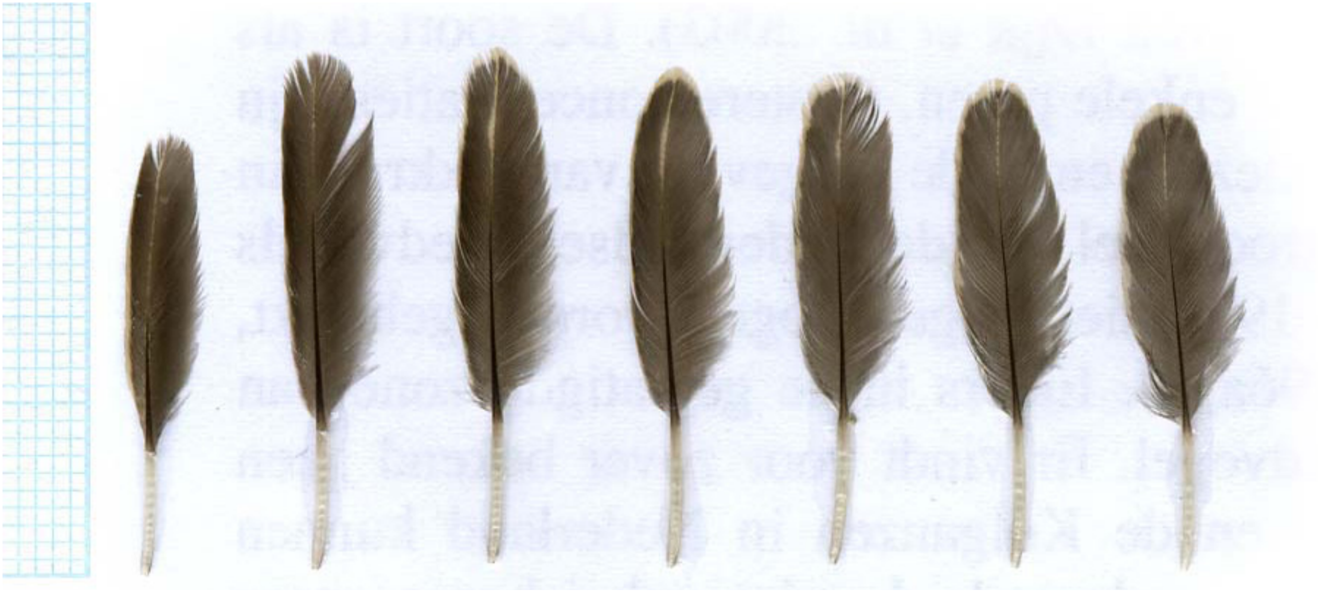

7. Supplementary Figure S7. UMPC tract from the adult Common Quail (*Coturnix coturnix*) specimen studied in Kaye *et al.* 2019[6]. Right wing; distal is towards the left. Note the centerlines lacking S-curves. Scale bar increments: 1 mm. Original image hosted at http://michelklemann.nl/verensite/coturnix_coturnix/cocoa04r.jpg[9].

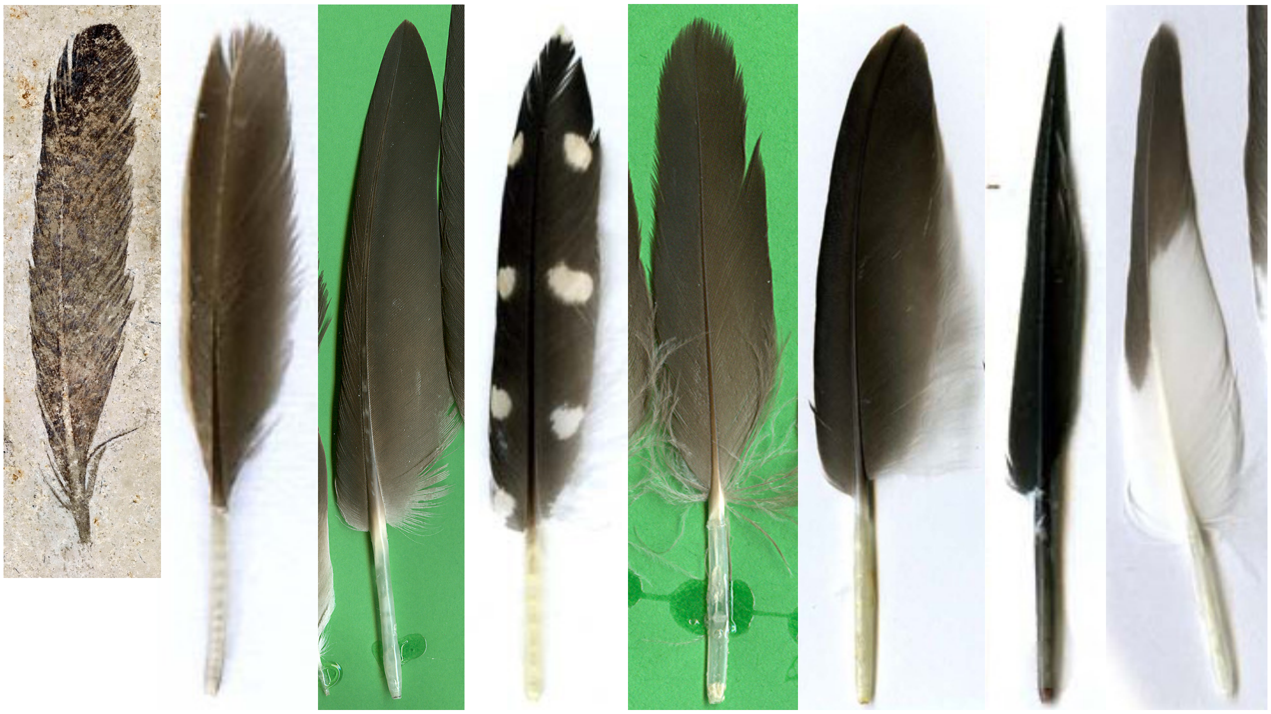

8. Supplementary Figure S8. Various UMPCs without S-shaped centerlines, from the same specimens and/or species studied in Kaye *et al.* 2019[6], compared with the isolated fossil feather. From left to right: Berlin slab (MB.Av.100)[7], Common Quail (*Coturnix coturnix*)[9], Common Crane (*Grus grus*)[5], Giant Kingfisher (*Megaceryle maxima*; VIII')[9], Long-tailed Duck (*Clangula hyemalis*; II')[5], Peregrine Falcon (*Falco peregrinus*; VIII')[9], Common Swift (*Apus apus*; X')[9], and Black-headed Gull (*Larus ridibundus*; X')[9]. Feathers not to scale; some images are reversed for consistency. Higher-resolution images of the Long-tailed Duck and Common Crane used with the permission of Stephan Schubert.

Anatomical identity

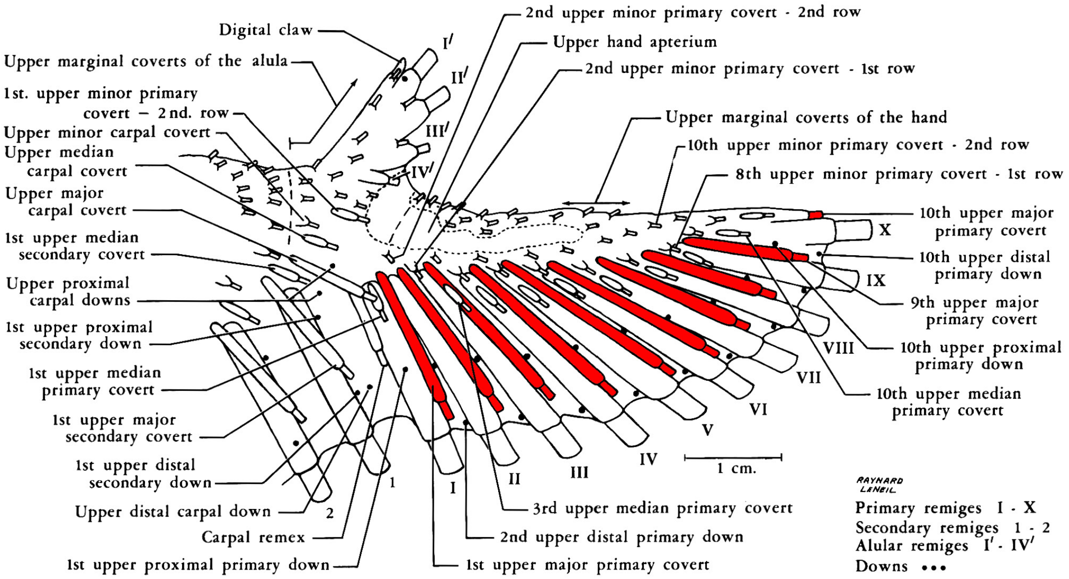

9. Supplementary Figure S9. Dorsal surface of the right manus of the chicken, *Gallus gallus domesticus*, modified from Lucas & Stettenheim 1972: Fig. 69[10]. Note the close and parallel attachment of the UMPC calami (red) with the respective primary calami. See also Hieronymus 2016[11].

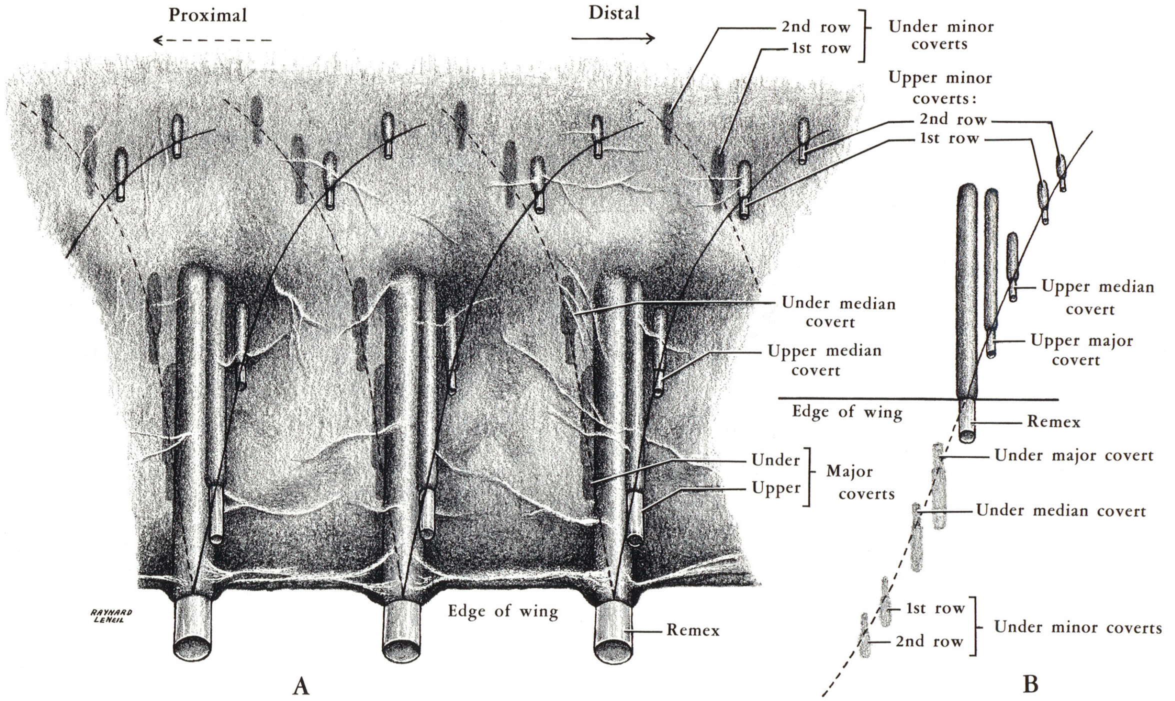

FIGURE 72.—Arrangement of the rows of coverts on the upper and under surface of hand and forearm. Lines join the rows of coverts with the remiges.

A, coverts are shown on a wing represented as semitransparent. The rows on the upper surface are inclined distally, and those on the under surface are inclined proximally.

B, illustrates the continuity that would exist if the surfaces could be laid out in a single plane.

10. Supplementary Figure S10. Schematic of calami, modified from Lucas & Stettenheim 1972[10]. Note the relatively long calamus of the upper major covert (**B**), attached closely to and in parallel with the respective primary calamus (**A**).

11. Calamus preservation. Freshly quarried fossils from the Solnhofen limestone may show faint outlines of structures from delicate soft parts for a short period of time (hours to weeks), which become invisible when the stone has dried out (H.T. personal observation). Thus, in addition to the initially faint calamus of the isolated fossil feather, it is also possible that the calamus outline faded shortly after von Meyer's illustration. This is plausible given that von Meyer had received the feather specimen directly from the quarry[8]. It is unknown when exactly von Meyer made his illustration, which occurred sometime between the fossil's discovery in spring or early summer 1861, and the publication in April 1862[12,13], although no illustration or mention thereof is made in von Meyer's letters from August and September 1861[14,15]. Regardless, by the time de Beer (1954)[16] published photographs of the two feather slabs, no such calamus was visible on either. Degradation of any visible calamus was likely exacerbated by exposure and handling since its discovery (e.g., note the fingerprints on the Munich slab's feather[6,17]). Some decades later, the Berlin slab (Supplementary Fig. S1) was further prepared, and any visible calamus traces that remained may have been removed at this time. Indeed, Griffiths (1996)[18] explicitly mentions damage to the fossil due to "poor preparatory technique," and scratch lines are clearly visible on the calamus region in his Plate 5, Fig. 1. The missing calamus itself wasn't specifically noted until 2002, and was attributed to wear[19].

12. Calamus measurements. Measurements of the isolated fossil feather were taken according to Lucas & Stettenheim 1972[10] (p.277):

> "There are several dimensions of feathers, the measurements of which can help to identify them as to the kind of bird or the tract where they originated. One or more of these measurements is included in each of the descriptions of feathers in this chapter. The dimensions, as we have measured them, are as follows:
> *Length*.—The length is measured from the bottom of the calamus to the tip of the vanes, with the shaft as straight as possible and the terminal barbs extended as far as possible.
> *Width*.—The width is the maximum distance across both vanes with the barbs at their normal angles from the rachis. In flight feathers and their coverts, it may be necessary to measure the widths of the vanes separately.
> *Calamus length*.—The length of the calamus is measured from the rim of the superior umbilicus, as close as possible to the midventral line (depending on the structure of the afterfeather), to the proximal end."

Given that the superior umbilicus is not visible in the fossil, the lowermost barbs were used as the distal limit of the calamus (Ibid., p.235):

> "The division between the calamus and the rachis is marked by the lowermost barbs and by a small opening to the calamus, the superior umbilicus (*umbilicus superior*, umbiliciform pit) on the ventral surface of the shaft (fig. 159)."

Relative calamus length refers to calamus length divided by total feather length (see also *15. Anatomical attributes*). To account for the curvature in the isolated fossil feather and replicate the aforementioned measurements "with the shaft as straight as possible", the curvilinear lengths of the calamus and rachis were used–as if the fossil were a modern feather that could be straightened out. Some additional notes on the three landmarks used for these measurements:

1). Distal tip (Supplementary Fig. S11a–d). In von Meyer's two descriptions of the feather, he noted that "The end of the vane is somewhat obtusely angled."[14], and that this distal tip had a "blunted end" that was "less rounded, more angularly formed than in the living Grey Partridge."[8] However, he drew the distal tip (a) as somewhat more rounded than it appears in the two slabs. In the Berlin slab (b), some damage is visible at the barb edges, due to the aforementioned preparatory scratches[17,18] (Griffiths 1996: Plate 6[18]). In the Munich slab (d), the feather shows a slightly sharper distal leading edge and tip than in the Berlin slab.

2). Calamus-rachis demarcation (Supplementary Fig. S11e–h). The feather on the Berlin slab was coated with varnish, which artificially thickened the appearance of barbs at the base[17] (f).

3). Proximal tip (Supplementary Fig. S11i–l). Given that the proximal portion of the calamus is not visible in the present-day Berlin and Munich slabs, the only evidence of its morphology is von Meyer's original mirror trace[8] and the recent laser-stimulated fluorescence (LSF) image[6]. In the former, no distinct border was drawn for the proximal tip (j). As von Meyer noted, "The feather is excellently preserved; only the end of the quill is less clearly expressed"[8]. The proximal tip is indistinct in the LSF image as well (l). Albeit speculative, the light grey region immediately below our reconstructed calamus (k) suggests the possibility that the proximal tip may have been slightly longer, perhaps representing a more gradual tapering to the inferior umbilicus. If so, our current measurement of relative calamus length would be an underestimation.

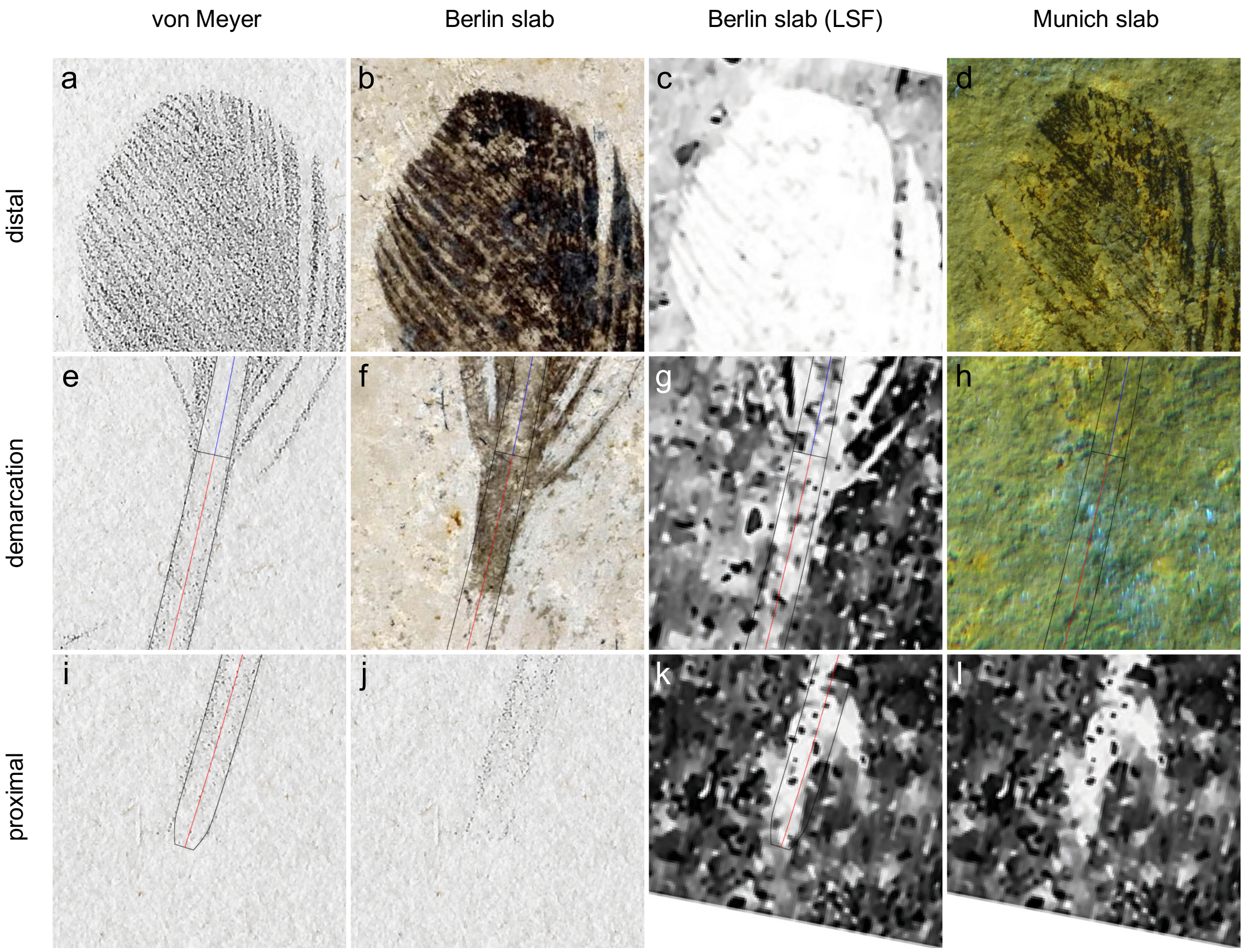

13. Supplementary Figure S11. Comparative images of the isolated fossil feather. The top and middle rows illustrate the distal tips and calamus-rachis demarcation, respectively, of the von Meyer reconstruction (**a,e**)[8], Berlin slab (reversed) under visible light (**b,f**)[7] and LSF (**c,g**)[6], and the Munich slab under ultraviolet (UV) light (**d,h**). The bottom row illustrates the proximal tips of the von Meyer reconstruction (**i,j**)[8] and LSF image (**k,l**)[6]; no proximal tips are observable in the Berlin and Munich slabs under visible and UV light. Black lines represent the calamus/rachis demarcation and outline, blue line represents the rachis portion of the centerline measurement, and red line represents the calamus portion of the centerline measurement. Information from the four image sources above, as well as from Griffiths 1996: Plate 6[18], is incorporated into our composite reconstruction of the feather (Supplementary Fig. S17). Panels: 1 cm x 1 cm.

a

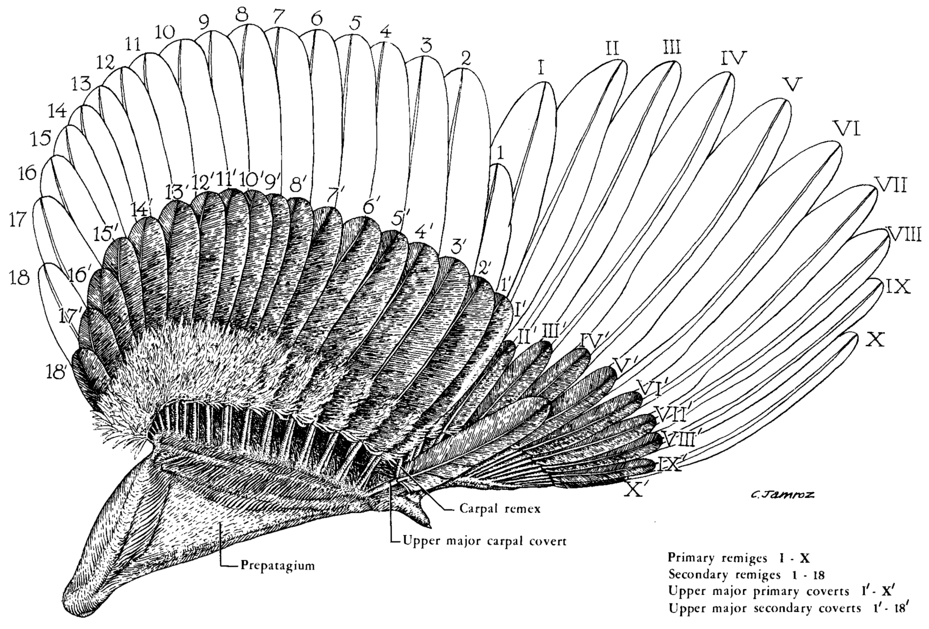

b

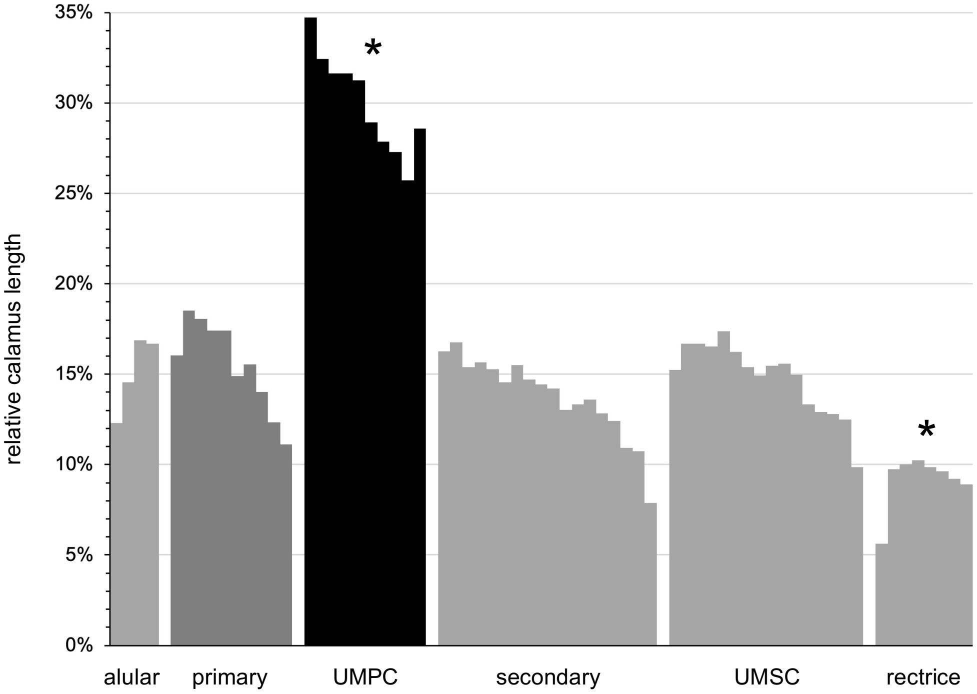

14. Supplementary Figure S12. Wing feathers and relative calamus lengths from the chicken, *Gallus gallus domesticus*. (**a**) Anatomical reference illustrating dorsal view of the left wing, reproduced from Lucas & Stettenheim 1972: Fig. 113[10]. UMPC I' is hidden in the drawing; alular feathers are not shown. Primaries and their respective coverts are numbered proximodistally using Roman numerals, and all major coverts are denoted with an apostrophe. (**b**) Relative calamus lengths grouped by tract, and ordered by ascending feather number. Colour coding follows Fig. 2a. The relative calamus lengths of the UMPCs (n = 10) and rectrices (n = 8) are significantly longer (P-value < 0.0001) and shorter (P-value < 0.001) than each of the other five tracts, respectively (asterisks; see Methods). Relative calamus lengths were calculated using published measurements from Lucas & Stettenheim 1972[10].

15. Anatomical attributes. See schematic in Fig. 2b. Table 1 includes designations that are generalized to extant birds, whereas Tables 2 and S1 include designations specific to three *Archaeopteryx* specimens. Attributes are assessed independently within a tract. Therefore, not all designations for a tract may apply to a single feather, and a single designation may not apply to all feathers within a tract. Both circumstances are particularly true in the case of "maybe" (occasionally). Numerous "maybe" designations thus have a multiplicative effect, further increasing the improbability of such tracts as a potential match for the isolated fossil feather (e.g., primaries in Table 1).

#1. Relative calamus length refers to calamus length divided by total feather length (see also *12. Calamus measurements*). Values from Fig. 2a and Supplementary Fig. S12 serve to quantitatively assess the relative calamus lengths across feather tracts in one extant specimen, and are not meant to be interpreted as strict absolute limits across all of avian diversity. Specifically, after the relative calamus lengths of the UMPC tract, those of the primary feathers (n = 10, range = 11.1–18.5%; dark grey) are the next closest match to that of the isolated feather (23.3%). In certain species, the relative calamus lengths of primaries can be as long as that of the isolated feather, or even too long. For example, in large flying birds such as swans, the relative calamus length of distal primaries can exceed 30%[10]. Therefore, in Table 1 we designate the relative calamus length for primaries as "maybe". All other feather tracts are considered ruled out by the relatively long calamus of the isolated feather (Fig. 2a); see also Supplementary Figs. S9, S10. While uMPCs and their calami were not individually measured by Lucas & Stettenheim 1972[10], the authors did illustrate such calami (Supplementary Fig. S10; see also Supplementary Fig. S5b) and state that these coverts "can be distinguished from the upper major primary coverts by the shorter calamus." Thus, we designate the uMPC tract as "no". The calamus-rachis demarcation was not observable in any of the major primary coverts preserved in the skeletal specimens of *Archaeopteryx*.

#2. Length refers to total feather length, from the proximal tip of the calamus to the distal tip of the vanes ("with the shaft as straight as possible and the terminal barbs extended as far as possible"[10]). It should be noted that there are exceptions to the general rule of certain modern and *Archaeopteryx* feather tracts being too long (compared with the isolated feather), often with exclusionary tradeoffs. For example, "maybe" is designated for the *Archaeopteryx* distal primaries and secondaries (Table S1), given the few distalmost and proximalmost feathers being exceptionally shorter than the rest of the respective tracts (Fig. 4). However, for each of these particular feathers of appropriate length, the width and/or vane asymmetry is uniquely inconsistent with that of the isolated feather.

#3. Width refers to the maximum distance across both vanes. This is a relatively uninformative attribute, given that the width of the isolated feather is potentially consistent with that of all nine tracts of modern feathers. This attribute does however exclude the narrow-vaned contours observable in the Altmühl and Berlin specimens of *Archaeopteryx*.

#4. Aspect ratio refers to an individual feather's length (#2) divided by width (#3). Given that all three attributes are assessed independently, tracts (and/or individual feathers) may be designated "no" for one attribute but "yes" for another. For example, Table S1 contains three tracts designated "no" for aspect ratio, despite designations of "maybe" for length and "yes" for width. In total, aspect ratio eliminates six tracts in *Archaeopteryx*, including the primaries and secondaries.

#5. Lateral curvature refers to a posterior curve of the centerline, towards the feather's trailing edge (not to be confused with the animal's anatomical "lateral"). This is differentiated from an S-shaped centerline, which has an inflection point that curves the rachis towards the feather's leading edge. As discussed by Norberg[20,21], flight feathers are curved backwards to increase dorsoventral stiffening, and to permit nose-up rotation during upstroke in order to let air through the wing.

#6. Barb angle refers to the angle between a barb and its rachis. Given the vast amount of variation in barb angles among extant bird species, the presence of barb angle asymmetry was used to assess consistency with the isolated feather (i.e., more acute barb-rachis angles on the leading vane compared with those on the trailing vane[22]). Given the more tractable sample size of *Archaeopteryx* specimens used in Tables 2 and S1, both the presence of asymmetry and measurements of barb angles were used to assess consistency with the isolated feather (see also Supplementary Figure S15). Functionally, barb angle in part determines the vane width and flexibility[10,23].

#7. Vane asymmetry refers to the narrower width of the leading vane compared with that of the trailing vane[10]. Vane asymmetry in the isolated feather was mentioned by von Meyer in his initial description[14], and measured by Speakman & Thomson 1994[24] as 2.2 at 25% distance from the feather tip. In modern birds, vane asymmetry is usually found in flight feathers and their coverts, and in some tail feathers[10,25]. This attribute is associated with aerodynamic function, by stiffening the narrower leading vane[25], and enabling rotation about the longitudinal feather axis during flapping flight[20,21].

#8. Vane closure refers to the closed pennaceous vanes that are provided by interlocking barbules (e.g., Supplementary Fig. S16), and that allow for feathers to function as a coherent aerodynamic surface[10,22,26].

#9. Angled distal tip refers to the leading and trailing edges of the vanes meeting at an angle, as opposed to forming an entirely rounded tip. Previous authors have also referred to the former condition as "blunted"[8], "clipped"[18], or "pointed"[10]. The presence and morphology of this attribute can vary along a feather tract (e.g., Supplementary Fig. S6). Ontogenetic variation and sexual dimorphism of this attribute in coverts was also reported in Lucas & Stettenheim 1972[10]. Alular feathers can have angled distal tips as well[5,9] (not included, since they are not present in *Archaeopteryx*). An angled distal tip helps to increase aerodynamic lift by reducing tip vortices[18].

Fossil evidence

feather tract	1. Relative calamus length	2. Length	3. Width	4. Aspect ratio	5. Lateral curvature[20,21]	6. Barb angle[35,37]	7. Vane asymmetry [18,20,21,24,25]	8. Vane closure	9. Angled distal tip[18]
**UMPC**[7]	?	**yes**	**yes**	**yes**	**yes**	**yes**	**yes**	**yes**	?
uMPC	?	**yes**	**yes**	**yes**	**yes**	**yes**	**yes**	no	no
**primary, distal**[18,53]	?	maybe[A,B]	**yes**	no	maybe[A,B]	no	**yes**	**yes**	no
primary, proximal	?	no	**yes**	no	no	**yes**	**yes**	**yes**	no
**secondary**[12,13,18,20]	?	maybe[A,B]	**yes**	no	maybe[L]	**yes**	**yes**	**yes**	no
UMSC	?	maybe[A]	?	?	no	**yes**	?	?	?
rectrice, outer	?	maybe[B,L]	**yes**	no	no	**yes**	**yes**	**yes**	no
rectrice, inner	no	no	**yes**	no	no	no	no	**yes**	no
**contour**[6]	?	no	no	no	**yes**	no	no	**yes**	no

16. Supplementary Table S1. Comparison of anatomical attributes shared by the isolated fossil feather and feather tracts of *Archaeopteryx*, based on the Altmühl*, Berlin[54], and London specimens. Rows ranked according to Table 1. Consistency between the isolated feather and a given feather tract is designated "yes," "no," "maybe" (occasionally; superscript letters denote relevant specimens), or "?" (not observable) (see *15. Anatomical attributes*). Superscript numbers denote additional references for individual attributes (column headings), as well as previous hypotheses of anatomical identity (boldface row headings). Abbreviations: UMPC, upper (dorsal) major primary covert; uMPC, under (ventral) major primary covert; UMSC, upper major secondary covert. **Note that elongate contours of the body and hindlimb[55] do exhibit lateral curvature (#5) and vane closure (#8), but are nonetheless inconsistent with the isolated feather with respect to all remaining observable attributes.*

17. Other *Archaeopteryx* specimens. Primary coverts are not observable in most of the known skeletal remains[12,13], including the Haarlem (TM 6928 / 6929), Eichstätt (JM 2257), Solnhofen (BMMS 500), Daiting (8th), Ottmann & Steil (9th, aka. Bürgermeister-Müller), Schamhaupten[27] (12th), and Mühlheim[28] (13th) specimens. Very faint impressions of possible primary covert rachises are visible in the Maxberg (see below), Munich (BSP 1999 I 50), and Thermopolis (WDC-CSG-100) specimens. While such impressions are anatomically uninformative, those of the primary coverts on the Thermopolis specimen's left wing do suggest a posterior orientation (also described for the secondary coverts[29]) consistent with that of the Altmühl and Berlin specimens' primary coverts (see main text).

In the Maxberg specimen–the original of which has been lost since 1991–the wing feathers remained firmly attached[12,13]. We can infer that both surfaces of the wings are present, given that the left and right manus are in dorsal and palmar views on the main slab, respectively. On the counterslab, a furrow is visible on a partial rachis impression from a primary on the left wing, indicating the ventral surface (Supplementary Fig. S13c). In the initial descriptions, Heller[30-32] identified numerous impressions covering the quills of the primaries that undoubtedly represent the primary covert feathers. However, the primary covert impressions we observed from photographs of nine casts of the main slab ($n = 5$) and counterslab ($n = 4$) were not distinct enough for assessing any anatomical attributes.

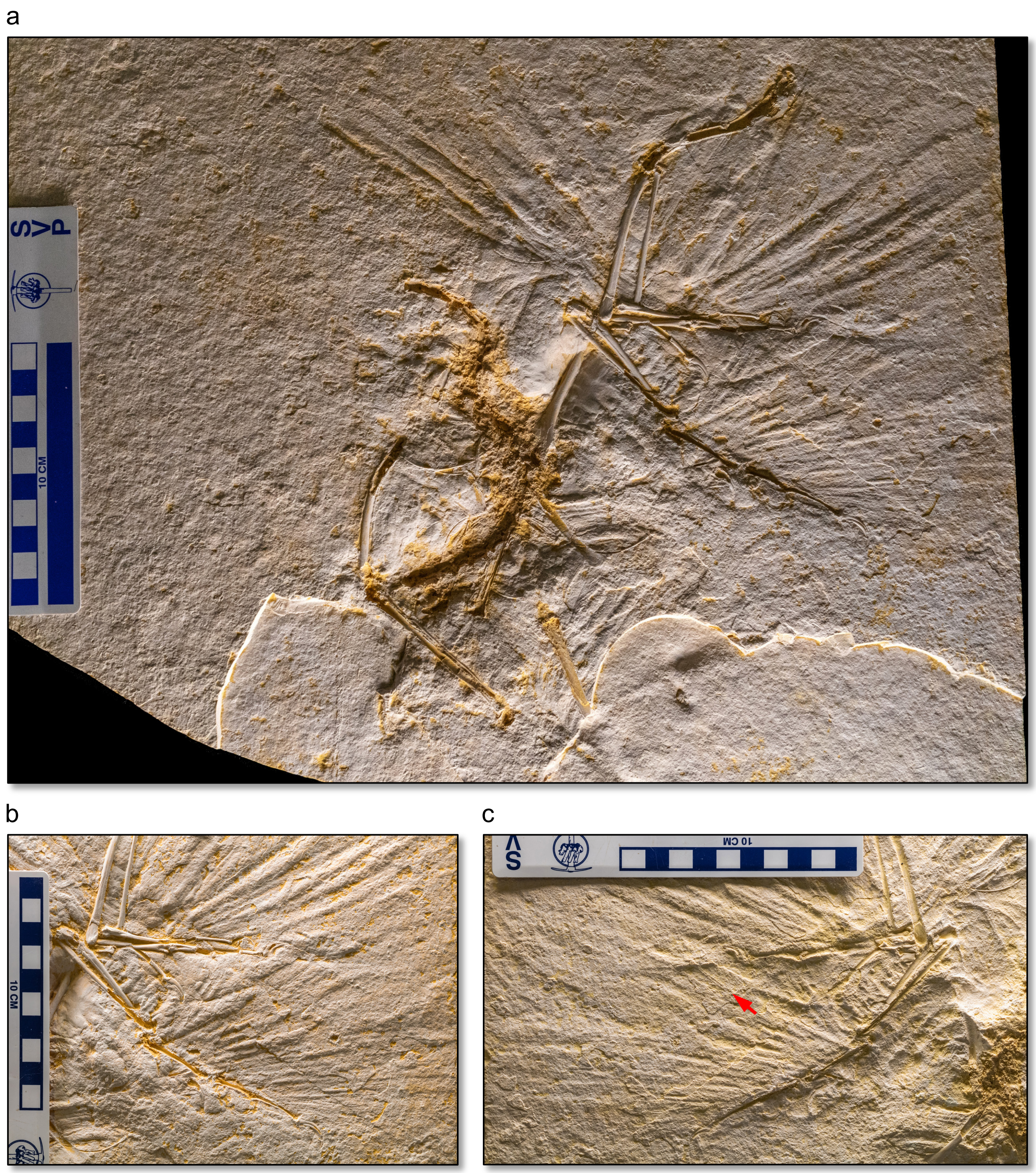

18. Supplementary Figure S13. Photographs of the Maxberg specimen of *Archaeopteryx* under oblique lighting, including coloured casts of the main slab (**a**,**b**; YPM VP.009807.002) and counterslab (**c**, YPM VP.009807.001). Arrow in (**c**) denotes a ventral furrow on a primary rachis from the left wing. Photographs courtesy of Jamie Henderson.

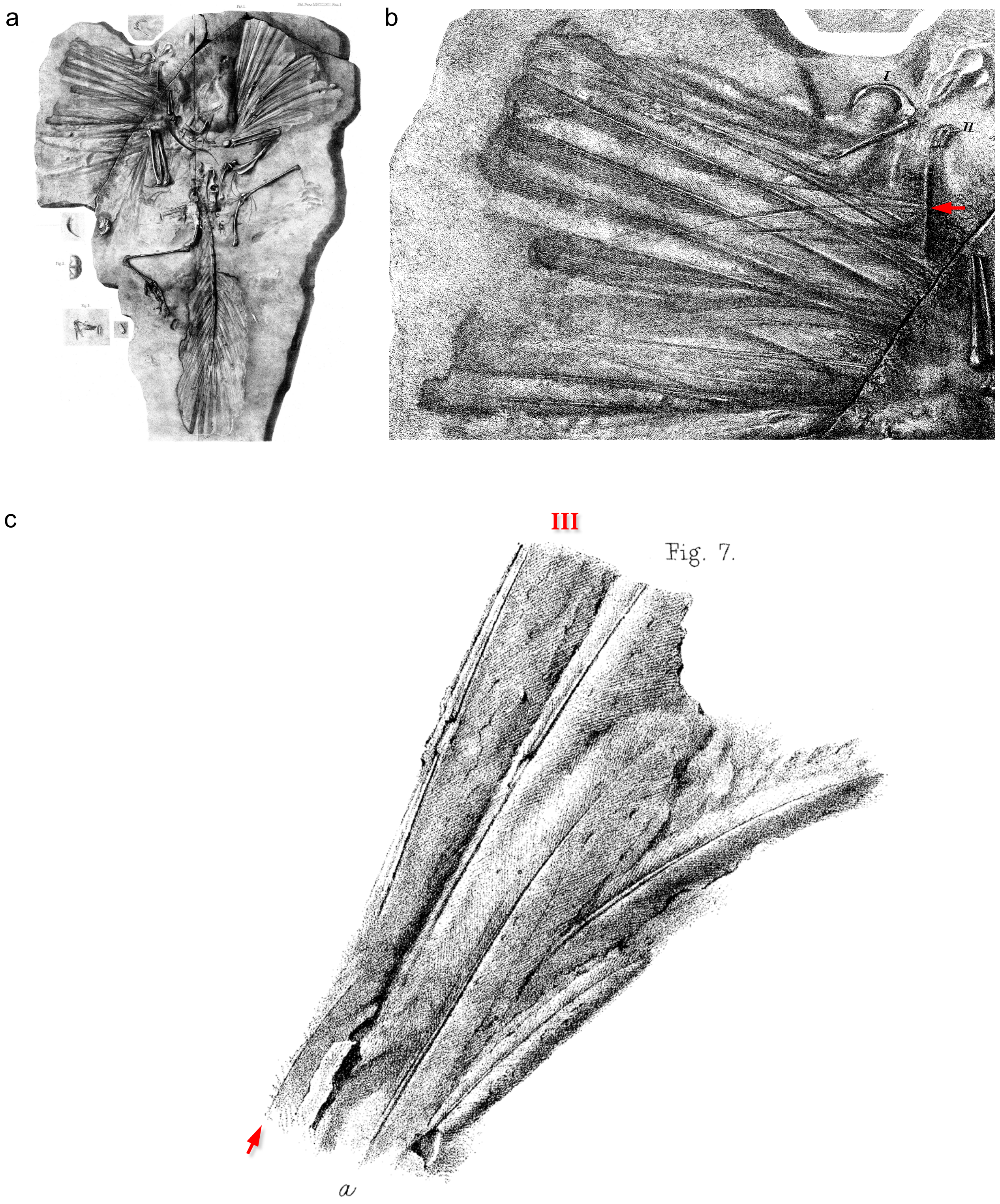

19. Supplementary Figure S14. London specimen of *Archaeopteryx* (neotype, NHMUK PV OR 37001). Reproduction of illustrations from Owen 1863: Plate I, Fig. 1; Plate IV, Fig. 7[33], showing the underside of the wings on the main slab. (**a**) Entire specimen and (**b**) left wing. Note the conspicuous uMPC centerline impression (to the left of the red arrow) discussed in the main text (Fig. 5ef), emanating from digit II phalanx 2. (**c**) Right wing. The small feather at the bottom left (red arrow) was misidentified as UMPC II' by Steiner 1918[34], and may represent uMPC III' (respective primary III labeled above, per de Beer 1954[16]). Feather "*a*" is possibly a regrown (post-moult) primary I[16], and not a covert (contra Owen 1863[33])[34].

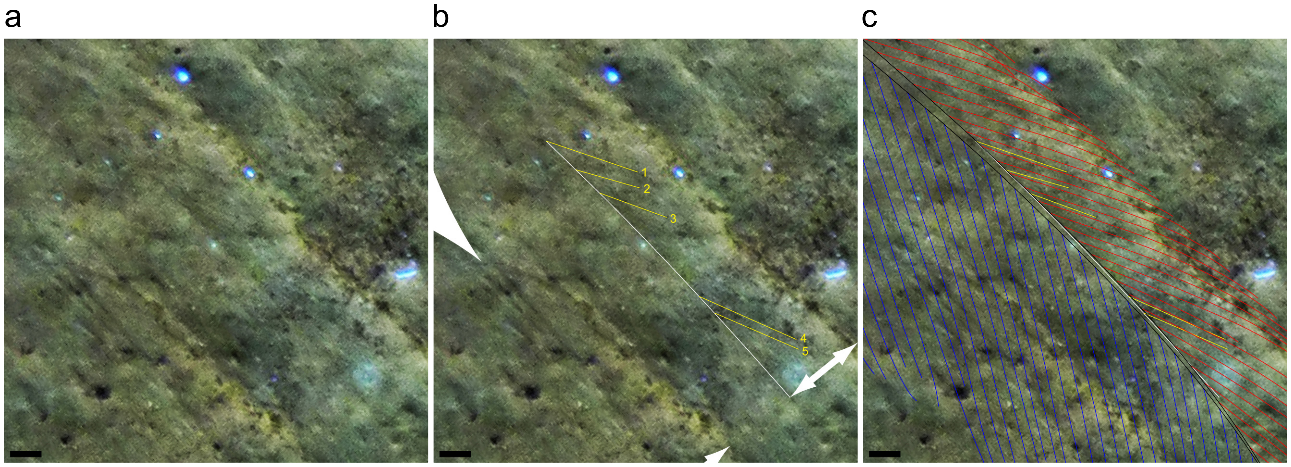

d

	**Altmühl** (UMPC; **b**)	**isolated feather** (**c**)	**London** (uMPC; not shown)
	-	25.8°	24.6° (Fig. 5)
	25.3° (**1**)	24.0°	
	27.8° (**2**)	26.5°	
	25.1° (**3**) (Fig. 4)	25.2°	
	23.1° (**4**)	24.3°	
	24.8° (**5**)	22.2°	
mean (*n* = 5):	25.2°	24.4°	
mean (*n* = 6):	-	24.7°	

20. Supplementary Figure S15. Corresponding barb angle measurements from leading vanes. (**a**) Photograph of UMPCs in the Altmühl specimen of *Archaeopteryx* under UV light. (**b**) Overlay of the rachis impression (white line) and barb angle measurements (numbered yellow lines, corresponding to (**d**)). Large arrow (neighbouring rachis) and double arrows (leading vane widths) correspond to those of Fig. 4. (**c**) Overlay of the isolated feather, per Fig. 4; leading vane barbs are shown in red. (**d**) Barb angles measured from coverts in the Altmühl (**b**) and London (Fig. 5) specimens, and the spatially corresponding barb angles (based on location of attachment) measured from the isolated feather (**c**). The close similarity in values is remarkable given the variation in barb angles–especially those of the leading vane–exhibited among different feathers and species[23,35–37]. Our measurements from the isolated feather are also consistent with the general barb angle of "approximately 25 degrees" reported for the isolated feather by Griffiths 1996[18]. Scale bar: 1 mm.

Additional insights

21. Designation. Previous interpretations of the Munich slab (BSP 1869 VIII 1) as the main slab (e.g., de Beer 1954[16]) may have been due to that half's accessioning prior to the Berlin slab (1869 vs. 1876[12,13]), and/or the fact that von Meyer illustrated the Munich slab's outline and feather chirality (1862: Plate VIII, Fig. 3)[8]. However, von Meyer had access to both slabs[8], and it has been hypothesized that he illustrated the fine details of the much better-preserved feather of the Berlin slab, by using a drawing mirror[38,17]. Given that this would yield a reflection that matches the feather on the Munich half, it makes sense that von Meyer would then draw or trace the outline of the Munich slab instead of the Berlin slab. Additionally, while von Meyer refers multiple times to the two halves as "both counterslabs"[14,8], he also refers to the thicker Berlin half as "the slab most clearly reproducing the feather... This slab is 0.022, the counterslab 0.016 [meters] thick."[8]

22. Feather chirality. While the Berlin slab's originally darker feather trace may be due in part to taphonomic effects (or subsequently, varnish; Tischlinger & Unwin 2004[17]), it may also reflect a natural differential in pigmentation. In the wing feathers of modern birds, the exposed surface is generally more melanized than the unexposed surface, for both visual and structural reasons[10,39]. If this were also the case in the fossil feather, the Berlin slab would thus represent the dorsal surface of the feather (more accurately, a mirror image imprint of it). This dorsal imprint interpretation is corroborated by microstructural evidence of the barbules, which Carney *et al.*[7] found to be "indistinguishable from those of modern pennaceous feathers with respect to morphology (Lucas & Stettenheim 1972) and barbule angles (Carlisle 1925)". In modern birds, feather barbicels always pass dorsally over the proximal barbules (Supplementary Fig. S16f)[10,40]. In the Berlin slab, the fact that the preserved barbicels lie underneath the proximal barbule (Supplementary Fig. S16c) reveals that we are observing the ventral surface of the feather microstructure (with the dorsal surface facing into the matrix). Thus, the darker and dorsal imprint of the Berlin slab, oriented by the narrower leading vane, indicates that the feather originated from the left wing of the animal. This left wing designation would likely hold true even if the feather were an under major or under median covert, as these "reversed coverts" have the same dorsoventral orientation as the upper coverts in modern birds (unlike the under *minor* coverts)[4,41-43].

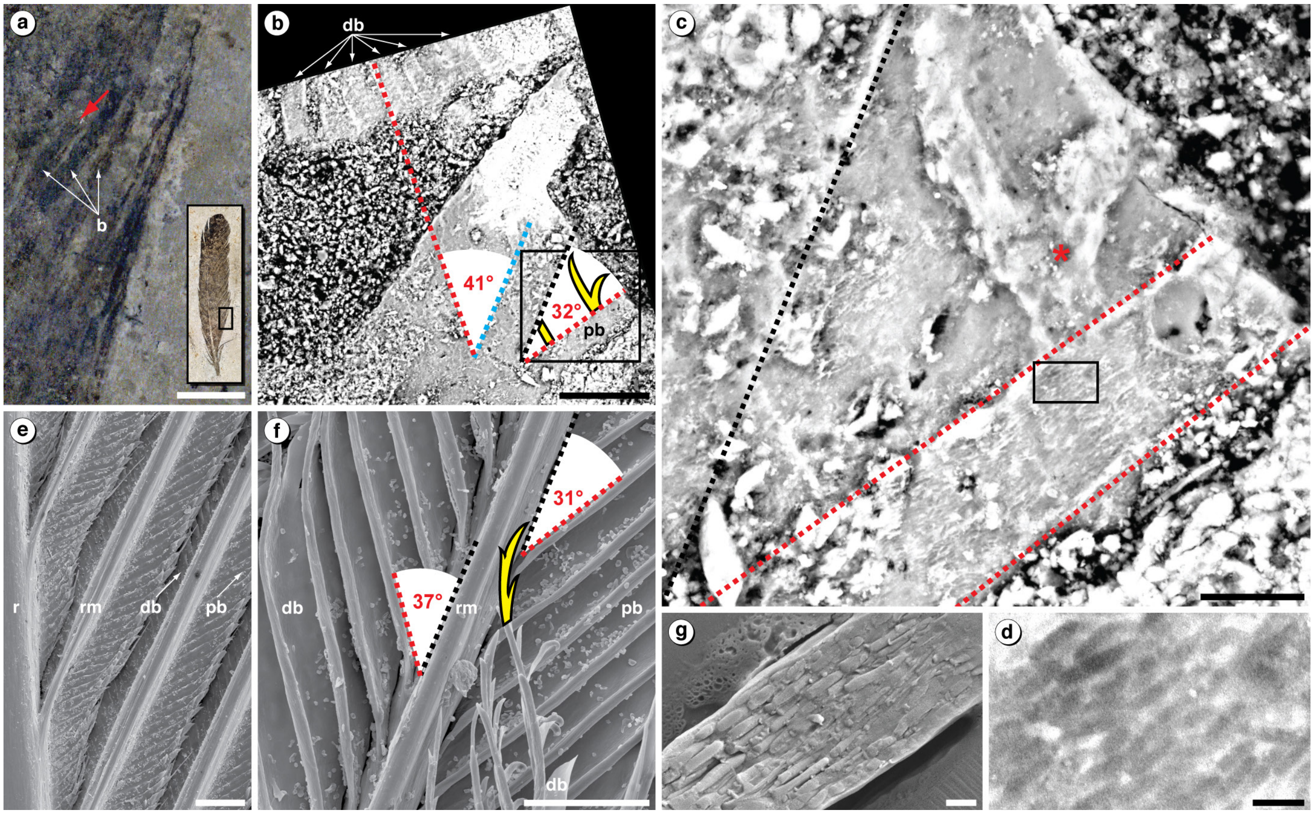

23. Supplementary Figure S16. Barbule microstructure in the Berlin slab of the isolated feather (MB.Av.100) and extant bird feathers; image and legend modified from Carney *et al.* 2012: Fig. 3[7]. (**a**) Optical photograph of boxed area within the trailing vane of the isolated feather (inset), illustrating individual barbs (white arrows). Scale bar: 1 mm. (**b**) SEM image from (**a**, red arrow) showing topographic impressions of a parallel array of distal barbules (db) and a proximal barbule (pb; boxed area), all of which contain melanosome imprints. (**c**) Detail of boxed area in (**b**). In (**b**) and (**c**), dashed red lines indicate ridges delineating the margins of barbules. Note that the proximal barbule overlays the preserved barbicels (**b**, in yellow; **c**, red asterisk), providing evidence that we are observing the ventral surface of the feather microstructure (see *22. Feather chirality*). Dashed black line represents possible ridge delineating the proximal margin of the corresponding barb ramus. In (**b**), the reconstructed distal margin of the ramus (blue dashed line) is not based on a preserved structure, but rather drawn parallel to the preserved proximal margin. Both distal and proximal barbule angles measured in the isolated feather (**b**) are very similar to those of the modern UMPC feather in (**f**). All these values are also consistent with the extensive barbule angle measurements of Carlisle 1925[40], which measured primary feathers from 62 species and 17 orders of birds, and found mean angles for distal barbules to be 41.5° (range 29°–58°) and proximal barbules to be 28.4° (range, 10°–41°). Scale bars: 50 μm (**b**) and 10 μm (**c**). (**d**) Detail of boxed area in (**c**), showing rod-shaped eumelanosome imprints aligned along the longitudinal axis of the barbule. Scale bar: 1 μm. (**e**,**f**) SEM images of barbs from a primary covert of the American Woodcock (*Scolopax minor*), showing ventral and dorsal surfaces of the feather, respectively. In (**e**), note how the proximal barbules overlay the adjacent barb's distal barbules–indicative of the ventral surface of the feather, and equivalent to the pattern of overlap in the isolated fossil feather (**b**,**c**). Conversely, at the bottom of the enlarged view of the dorsal surface (**f**), the distal barbules and their hooklet-containing barbicels overlay the adjacent barb's proximal barbules. The terminal end of one of these barbicels is emphasized in yellow–equivalent to the barbicel structures in (**b**,**c**), but seen from the opposing feather surface. To the right, neighboring distal barbules have been removed to expose underlying proximal barbules. Scale bars: 100 μm (**e**) and 50 μm (**f**). (**g**) SEM image of longitudinal cross-section from a barbule of the White-breasted Nuthatch (*Sitta carolinensis*), showing the characteristic parallel alignment of melanosomes along the longitudinal axis of the barbule. Scale bar: 1 μm. Abbreviations: b, barb; db, distal barbule; pb, proximal barbule; r, rachis; rm, ramus.

24. Melanosomes. In Carney *et al.* 2012[7], the presence of melanosomes in the isolated *Archaeopteryx* feather was supported by detection of elongate microbodies identical to melanosomes in their 1). size, 2). shape, 3). limited morphological range, 4). parallel alignment, 5). absence of serial arrangement (cf. bacterial chains), and 6). location (within barbules), as well as by 7). corroborating evidence of colour patterns from other fossil feathers[44]. This conclusion was criticized by Moyer *et al.*[45], who recapitulated the obsolete interpretation[46,47] that such fossilized microbodies are microbes, due to similarities in size and shape. However, it should be noted that Moyer *et al.*'s comparisons with extant bacteria were actually dissimilar with respect to size[44], and invalid with respect to shape. Specifically, while the extant bacteria were shown in the same longitudinal view as the microbodies from the fossil feather (Moyer *et al.* 2014: Fig. 1d–f[45]), the extant melanosomes were inappropriately compared in cross-sectional view (Ibid.: Fig. 1b,c). The authors also state that in order to confirm the identity of microbodies in the fossil record, "molecular or chemical signals unique to either melanosomes or microbes in extant feathers" should be detected and "localized to the [fossilized microbody] structures to eliminate the alternative, using in situ surface techniques (e.g., time of flight secondary ion mass spectrometry (TOF-SIMS))". Yet, no such evidence was provided in support of their alternative microbe hypothesis. However, using TOF-SIMS, we subsequently detected 1). such molecular signals of melanin, 2). which were localized to melanosome-like microbodies 3). across a wide range of taxa, tissues, and environments–from the fossil feathers of the closely related Jurassic paravian *Anchiornis*[48] to the fossil skin of various marine reptiles[49]. Furthermore, 4). these molecular signatures were unique to animal eumelanin, and 5). no molecular signatures unique to microbes or microbial melanins were detected[48]. Together, this unequivocal and definitive molecular evidence of melanin and melanosomes in residues of fossil integument confirms our original interpretation, that the melanosome-like microbodies preserved in the isolated fossil feather are indeed melanosomes.

25. Colouration. The distinct black and white reconstruction of the isolated fossil feather in Manning *et al.* 2013: Fig. 1F[50] (reproduced below) was based on the distributions of organic sulphur and trace metals, used as putative biomarkers for eumelanin. However, while we agree that sulphur can diagenetically bind to eumelanin, sulphur also binds to other organic matter (sulphurization)[7]. The latter is evident in the paper's own Fig. 1D[50], where sulphur is bound to the plant matter surrounding the feather[18,12,13]. Similarly, the trace metals are abundant in the surrounding rock matrix of both the isolated feather (Manning *et al.* 2013: Fig. 1B,C[50]), and especially the counterslab of the Berlin skeletal specimen (Ibid.: Fig. 3). Thus, the true plumage patterns of *Archaeopteryx* remain unknown. Fundamentally, sulphur and trace metals are indirect proxies that are not specific to eumelanin[51], and therefore do not always indicate pigmentation patterns (false positive/type I error). By contrast, melanin residue and melanosomes are the actual pigment and pigment microstructures themselves, and therefore represent direct evidence of colouration. This direct evidence is also specific–here, the residue is constrained to the isolated feather structures and is not present in the surrounding plant matter or rock matrix[7]. Additionally, just because sulphur and trace metals are able to bind to eumelanin does not mean that they are *always* bound to eumelanin (false negative/type II error). Specifically, based on lower concentrations of copper, nickel, and organic sulphur (and speciously, a sulphate map), Manning *et al.*[50] reconstructed the feather's trailing vane as an "un-pigmented" white colour (below). However, white colour in feathers is produced by the absence of melanosomes[52]–therefore, if this area were truly white, there would be no melanosomes and certainly no melanin residue. Instead, this trailing vane is completely covered in dark melanin residue and contains thousands of observed melanosomes (e.g., Supplementary Fig. S16a–d). Thus, we reaffirm that the *Archaeopteryx* feather was *entirely* matte black, with a darker distal tip.

26. Supplementary Figure S17. Reconstructions from Manning *et al.* 2013: Fig. 1F[50] (left, reversed) and present study (right), at life size.

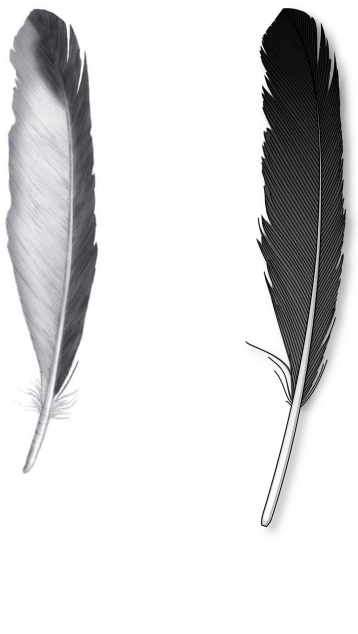

References

1. Pycraft, W. P. *A History of Birds*. Vol. 2. (Methuen and Company, 1910).
2. Jeikowski, H. Die Flügelbefiederung des Bleßhuhns (*Fulica atra* L.). *Journal für Ornithologie* **112**(2), 164–201 (1971).
3. Stegmann, B. Die verkümmerte distale Handschwinge des Vogelflügels. *Journal für Ornithologie* **103**(1), 50–85 (1962).
4. Wray, R. S. On some points in the morphology of the wings of birds. *Proceedings of the Zoological Society of London* **55**(2): 343–357, Plate XXXI (1887).
5. Schubert, S. *Bird Feathers*, http://www.vogelfedern.de/index-e.htm (2019).
6. Kaye, T. G., Pittman, M., Mayr, G., Schwarz, D. & Xu, X. Detection of lost calamus challenges identity of isolated *Archaeopteryx* feather. *Scientific Reports* **9**, 1182; https://doi.org/10.1038/s41598-018-37343-7 (2019).
7. Carney, R. M., Vinther, J., Shawkey, M. D., D'Alba, L. & Ackermann, J. New evidence on the colour and nature of the isolated *Archaeopteryx* feather. *Nature Communications* **3**, 637; https://doi.org/10.1038/ncomms1642 (2012).
8. von Meyer, H. *Archaeopteryx lithographica* aus dem lithographischen Schiefer von Solenhofen. *Palaeontographica* **10**(2), 53–56; Plate VIII, Fig. 3 (1862). (English translation, with redrawn figure: von Meyer, H. On the *Archæopteryx lithographica*, from the lithographic slate of Solenhofen. *Annals and Magazine of Natural History* **9**(53), 366–370 (1862).)
9. Klemann, M. *Feathers*, http://michelklemann.nl/verensite/start/featherrightindex.html (2019).
10. Lucas, A. M. & Stettenheim, P. R. *Avian Anatomy: Integument.* (U.S. Department of Agriculture, 1972).
11. Hieronymus, T. L. Flight feather attachment in rock pigeons (*Columba livia*): covert feathers and smooth muscle coordinate a morphing wing. *Journal of Anatomy* **229**(5), 631–656 (2016).
12. Wellnhofer, P. *Archaeopteryx. The Icon of Evolution.* (Verlag Dr. Friedrich Pfeil, 2009).
13. Wellnhofer, P. *Archaeopteryx. Der Urvogel von Solnhofen.* (Verlag Dr. Friedrich Pfeil, 2008).
14. von Meyer, H. Vogel-Feder und *Palpipes priscus* von Solenhofen. *Neues Jahrbuch für Mineralogie, Geognosie, Geologie und Petrefakten-Kunde.* **1861**, 561 (1861a).
15. von Meyer, H. *Archaeopteryx lithographica* (Vogel-Feder) und *Pterodactylus* von Solenhofen. *Neues Jahrbuch für Mineralogie, Geognosie, Geologie und Petrefakten-Kunde.* **1861**, 678–679 (1861b).
16. de Beer, G. *Archaeopteryx lithographica. A Study Based upon the British Museum Specimen.* (British Museum (Natural History), 1954).
17. Tischlinger, H. & Unwin, D. UV-Untersuchungen des Berliner Exemplars von *Archaeopteryx lithographica* H. v. Meyer 1861 und der isolierten *Archaeopteryx*-Feder. *Archaeopteryx* **22**, 17–50 (2004).
18. Griffiths, P. J. The isolated *Archaeopteryx* feather (Die isolierte *Archaeopteryx*-Feder). *Archaeopteryx* **14**, 1–26 (1996).
19. Elzanowski, A. Archaeopterygidae (Upper Jurassic of Germany). In: *Mesozoic Birds: Above the Heads of Dinosaurs* (eds. Chiappe, L. M. & Witmer, L. M.) 129–159 (University of California Press, 2002).
20. Norberg, R. Å. Function of vane asymmetry and shaft curvature in bird flight feathers; inferences on flight ability of *Archaeopteryx.* In: *The Beginnings of Birds: Proceedings of the International Archaeopteryx Conference, Eichstätt 1984* (eds. Hecht, M. K., Ostrom, J. H., Viohl, G. & Wellnhofer, P.) 303–318 (Freunde des Jura-Museums, 1985).
21. Norberg, R. Å. Feather asymmetry in *Archaeopteryx. Nature* **374**, 221 (1995).
22. Longrich, N. Structure and function of hindlimb feathers in *Archaeopteryx lithographica. Paleobiology*, **32**(3), 417–431 (2006).
23. Ennos, A., Hickson, J., & Roberts, A. N. N. A. (1995). Functional morphology of the vanes of the flight feathers of the pigeon *Columba livia. Journal of Experimental Biology* **198**(5), 1219–1228.
24. Speakman, J. R. & Thomson, S. C. Flight capabilities of *Archaeopteryx*. *Nature* **370**(6490), 514 (1994).
25. Feduccia, A. & Tordoff, H. B. Feathers of *Archaeopteryx*: asymmetric vanes indicate aerodynamic function. *Science* **203**(4384): 1021–1022 (1979).
26. Prum, R. O. Development and evolutionary origin of feathers. *Journal of Experimental Zoology* **285**, 291–306 (1999).
27. Rauhut, O. W. M., Foth, C. & Tischlinger, H. The oldest *Archaeopteryx* (Theropoda: Avialiae): a new specimen from the Kimmeridgian/Tithonian boundary of Schamhaupten, Bavaria. *PeerJ* **6**, e4191 (2018).
28. Rauhut, O. W. M, Tischlinger, H. & Foth, C. A non-archaeopterygid avialan theropod from the Late Jurassic of southern Germany. *eLife* **8**, e43789 (2019).
29. Mayr, G., Pohl, B., Hartman, S. & Peters, D. S. The tenth skeletal specimen of *Archaeopteryx*. *Zoological Journal of the Linnean Society* **149**(1), 97–116 (2007).
30. Heller, F. Ein dritter *Archaeopteryx*-Fund aus den Solnhofener Plattenkalken von Langenaltheim/Mfr. Erlanger Geologische Abhandlungen **31**, 1–25 (1959).
31. Heller, F. Der dritte *Archaeopteryx*-Fund aus den Solnhofener Plattenkalken das oberen Malm Frankens. *Journal für Ornithologie* **101**, 7–28 (1960).
32. Heller, F. & Stürmer, W. Der dritte *Archaeopteryx*-Fund. *Nature und Volk* **90**(5), 137–145 (1960).
33. Owen, R. On the *Archeopteryx* of von Meyer, with a description of the fossil remains of a long-tailed species, from the lithographic stone of Solenhofen. *Philosophical Transactions of the Royal Society of London* **153**, 33–47; Plate I, Fig. 1; Plate IV, Fig. 7 (1863).
34. Steiner, H. 1918. Das Problem der Diastataxie der Vogelflügels. *Jenaische Zeitschrift für Naturwissenschaft* **55**, 221–359 (1918).
35. Bachmann, T. *et al.* Morphometric characterisation of wing feathers of the barn owl *Tyto alba pratincola* and the pigeon *Columba livia*. *Frontiers in Zoology* **4**(1), 23 (2007).
36. Pap, P. L. *et al.* Vane macrostructure of primary feathers and its adaptations to flight in birds. *Biological Journal of the Linnean Society* **126**(2), 256–267 (2019).
37. Janda, V. Vergleichende Untersuchungen über den feineren Bau der Schwung-, Steuer- und kleineren Deckfedern bei wilden, domestizierten und künstlichen Einflüssen ausgesetzten Vögeln. *Zoologische Jahrbücher. Abteilung für allgemeine Zoologie und Physiologie der Tiere* **46**, 214–296 (1929).
38. Wellnhofer, P. Hermann von Meyer und der Solnhofener Urvogel *Archaeopteryx lithographica. Hermann von Meyer—Frankfurter Bürger und Begründer der Wirbeltierpaläontologie in Deutschland. Kleine Senckenberg-Reihe* **40**, 11–18 (2001).
39. Burtt Jr, E. H. An analysis of physical, physiological, and optical aspects of avian coloration with emphasis on wood-warblers. *Ornithological Monographs* **38**, 1–126 (1986).
40. Carlisle, G. C. 1925. Some observations on the base:pennulum ratio and angular ratio of the barbules of the primaries in various groups of birds. *The Ibis* **67**(4), 908–919 (1925).
41. Sundevall, C. J. On the wings of birds. *The Ibis* **28**(4), 389–457 (1886).
42. Bates, G. L. The reversed under wing-coverts of birds and their modifications, as exemplified in the birds of West Africa. *The Ibis* **60**(4): 529–583 (1918).
43. Humphrey, P. S. & Clark Jr, G. A. Pterylosis of the mallard duck. *The Condor* **63**(5), 365–385 (1961).
44. Vinther, J. Fossil melanosomes or bacteria? A wealth of findings favours melanosomes. *BioEssays* **38**(3), 220–225 (2016).
45. Moyer, A. E. *et al.* Melanosomes or microbes: testing an alternative hypothesis for the origin of microbodies in fossil feathers. *Scientific Reports* **4**, 4233 (2014).
46. Wuttke, M. "Weichteil-Erhaltung" durch lithifizierte Mikroorganismen bei mittel-eozänen Vertebraten aus den Ölschiefern der "Grube Messel" bei Darmstadt. *Senckenbergiana lethaea* **64**: 509–527 (1983).
47. Davis, P. G. & Briggs, D. E. G. Fossilization of feathers. *Geology* **23**(9), 783–786 (1995).
48. Lindgren, J. *et al.* Molecular composition and ultrastructure of Jurassic paravian feathers. *Scientific Reports* **5**, 13520 (2015).
49. Lindgren, J. *et al.* Skin pigmentation provides evidence of convergent melanism in extinct marine reptiles. *Nature*, **506**(7489), 484 (2014).
50. Manning, P. L. *et al.* Synchrotron-based chemical imaging reveals plumage patterns in a 150 million year old early bird. *Journal of Analytical Atomic Spectrometry* **28**(7), 1024–1030 (2013).
51. Vinther, J. A guide to the field of palaeo colour: melanin and other pigments can fossilise: reconstructing colour patterns from ancient organisms can give new insights to ecology and behaviour. *BioEssays*, **37**(6), 643–656 (2015).
52. Vinther, J., Briggs, D. E. G., Prum, R. O. & Saranathan, V. The colour of fossil feathers. *Biology Letters* **4**(5), 522–525 (2008).
53. Savile, D. B. O. The primaries of *Archaeopteryx. Auk* **74**(1), 99–101 (1957).
54. Rietschel, S. Feathers and wings of *Archaeopteryx*, and the question of her flight ability. In: *The Beginnings of Birds: Proceedings of the International Archaeopteryx Conference, Eichstätt 1984* (eds. Hecht, M. K., Ostrom, J. H., Viohl, G. & Wellnhofer, P.) 251–260; Plates 1, 2 (Freunde des Jura-Museums, 1985).
55. Foth, C., Tischlinger, H. & Rauhut, O. W. M. New specimen of *Archaeopteryx* provides insights into the evolution of pennaceous feathers. *Nature* **511**, 79–82 (2014).
